# Integration of Genetic and Immune Infiltration Insights into Data Mining of Multiple Sclerosis Pathogenesis

**DOI:** 10.1155/2022/1661334

**Published:** 2022-06-27

**Authors:** Xiaoyun Zhang, Ying Song, Xiao Chen, Xiaojia Zhuang, Zhiqiang Wei, Li Yi

**Affiliations:** ^1^Department of Rehabilitation, Shenzhen Longhua District Central Hospital, Shenzhen 518000, China; ^2^Department of Neurology, Peking University Shenzhen Hospital, Shenzhen 518000, China

## Abstract

**Background:**

Multiple sclerosis (MS) is an immune-mediated demyelinating disease of the central nervous system. MS pathogenesis is closely related to the environment, genetic, and immune system, but the underlying interactions have not been clearly elucidated. This study aims to unveil the genetic basis and immune landscape of MS pathogenesis with bioinformatics.

**Methods:**

Gene matrix was retrieved from the gene expression database NCBI-GEO. Then, bioinformatics was used to standardize the samples and obtain differentially expressed genes (DEGs). The protein-protein interaction network was constructed with DEGs on the STRING website. Cytohubba plug-in and MCODE plug-in were used to mine hub genes. Meanwhile, the CIBERSORTX algorithm was used to explore the characteristics of immune cell infiltration in MS brain tissues. Spearman correlation analysis was performed between genes and immune cells, and the correlation between genes and different types of brain tissues was also analyzed using the WGCNA method.

**Results:**

A total of 90 samples from 2 datasets were included, and 882 DEGs and 10 hub genes closely related to MS were extracted. Functional enrichment analysis suggested the role of immune response in MS. Besides, CIBERSORTX algorithm results showed that MS brain tissues contained a variety of infiltrating immune cells. Correlation analysis suggested that the hub genes were highly relevant to chronic active white matter lesions. Certain hub genes played a role in the activation of immune cells such as macrophages and natural killer cells.

**Conclusions:**

Our study shall provide guidance for the further study of the genetic basis and immune infiltration mechanism of MS.

## 1. Background

Multiple sclerosis (MS) is an immune-mediated demyelinating disease of the central nervous system (CNS) [1], which predominantly occurs in Caucasians and can cause severe neurological dysfunctions in advanced stages [2, 3]. The current knowledge has indicated that environmental and genetic factors jointly mediate the occurrence of MS. Although genes play an important role in the pathogenesis of MS, the concordant rate among identical twins was 30%, and a meta-analysis estimated that genetic factors accounted for only 50% of the risk of MS [4, 5], suggesting that nongenetic factors also greatly influence the susceptibility to the disease. Environmental risk factors such as vitamin D deficiency, smoking, and Epstein-Barr virus (EB virus) infection [6] can interact with genetic variations of MS and thus lead to immune dysregulation. However, the researches on the interaction between environment and gene are still in progress.

Peripheral immune cells, especially B cells and T cells, are implicated in the pathogenesis of MS. In the early stages of MS, the innate immune system dominated by macrophages promotes the proinflammatory response of *T* and B cells, leading to tissue damage. Early microglial activation may also be one of the initial events in the development of MS. Activated microglia contribute to disease progression by releasing proinflammatory cytokines, free radicals, and glutamate. In the progressive stages, pathological changes such as axonal degeneration, focal to diffuse white matter injury, microglial cell activation, lymphocyte diffusion, monocyte infiltration, and cortical involvement occur [7, 8]. On the one hand, immune cells are involved in promoting inflammatory response, demyelination, axon damage, and disease plaque formation. On the other hand, some immune cells also exert anti-inflammatory effects and inhibit disease progression by promoting tissue repair. The alterations of the immune system are complex in MS patients, and the contributions of immune cells to the pathology of MS have not been fully clarified.

Emerging studies on the subtypes of infiltrating immune cells in the brain of MS patients or experimental autoimmune encephalomyelitis (EAE) have revealed that both Th1 and Th17 CD4^+^ T lymphocytes are involved in disease initiation after in situ reactivation. However, the details of the immune microenvironment of intracranial lesions in MS patients remain unclear. Accumulating studies focus on the role of certain types of immune cells and their plasticity in the interaction with cytokines, chemokines, and brain compartments [9].

As early as 20 years ago, sequencing technology emerged, and gene chips contained a large number of biological information [10]. Databases of gene expression, gene polymorphism, protein structure, and genetic map are expanding. Gene microarray is an emerging technology that bears the advantage of an efficient and large-scale collection of gene expression profile data of diseases, which has been widely used in bioinformatics research [11]. A large amount of gene data has aroused the demand for bioinformatics [12]. In this backdrop, we can extract, process, and analyze microarray data more effectively with bioinformatic tools. With the emerging technique CIBERSORT, we can analyze the compositions of immune cells based on gene chips.

In the current study, combined with microarray data extracted from the existing MS samples, we used *R* language, STRING, and Cytoscape to calculate hub genes, annotate, and perform gene enrichment analysis. We used the CIBERSORT algorithm to infer the proportion of peripheral immune cells in brain lesions. Moreover, we calculated the correlation between hub genes and immune cells and used the weighted gene coexpression network (WGCNA) to further compare the internal gene differences in the dataset and analyze the correlation between hub genes and lesion sites. This study aimed to explore susceptibility genes and immune cell infiltration in brain lesions of MS patients, hoping to confer novel insights into MS prevention and treatment.

## 2. Methods

### 2.1. Differentially Expressed Genes (DEGs) Screening

Gene Expression Omnibus [13] (GEO; https://www.ncbi.nlm.Sal) is an open genomics database. Human expression profile datasets of MS (GSE108000 and GSE135511) were downloaded from the GEO database. Then, the datasets were divided into the control group and the MS group. And we used the normalizeBetweenArrays function in the *R* language “limma” package to normalize the datasets and eliminate intragroup differences. GSE108000 and GSE135511 were combined with “SVA” *R* package [14] to remove batches. DEGs with *p* < 0.05 and |log2FC| >0.5 were considered statistically significant. Based on the obtained DEGs, we used *R* package “ggplot2” [15] to draw a volcano plot to view the differences between upregulated and downregulated genes and used a heatmap to view the differences of genes between the disease group and control group.

### 2.2. Construction of Protein-Protein Interaction (PPI) Networks

STRING (https://string-db.org/) is a database for the study of PPI networks. The data are derived from published experimental data, and genes come from computer mining, prediction, and fusion. In our study, we used STRING 11.0 to analyze the DEGs obtained. The confidence score was set as 0.9. Since the regions with more interconnections in the PPI network have a higher probability of participating in the biological regulation, and the dissociated gene nodes seldom play a key role in the entire network, we chose to eliminate the dissociated gene nodes in our study. After that, we downloaded the network file for subsequent analysis.

Cytoscape is software for network analysis, which contains plug-ins such as Cytohubba, Molecular Complex Detection (MCODE), and ClueGo. Cytohubba can rank network nodes via various topological analysis methods. Among all the topological methods, the MCC method has the most accurate prediction value. MCODE can detect closely related regions in the PPI network and then infer different molecular complexes. With the Cytohubba plug-in and MCC method, we ranked network nodes and selected the top ten genes for further analysis. With the MCODE plug-in, we divided the network into different subnetworks and analyzed the first five subnetworks.

### 2.3. Enrichment Analysis

The commonly used enrichment analysis methods mainly include Gene Ontology (GO) [cellular component (CC), biological process (BP), and molecular function (MF)] and Kyoto Encyclopedia of Genes and Genomes (KEGG), which can reveal the functional tendency of gene sets. The commonly used enrichment tools include the DAVID database, KOBAS database, and key *R* package (clusterProfiler) [16]. The plug-ins include Funrich, ClueGo, Cluepedia, Metascape, etc. In our study, we used Cytoscape GlueGo (version 2.5.7) and Cluepedia (version 1.5.7) plug-ins to perform gene annotation analysis on the gene sets obtained from step 2 using MCODE and Cytohubba methods and used “Clusterprofiler” *R* package to analyze the DEGs obtained from step 1. The pathways with *p* < 0.05 and *Q* < 0.05 were screened out. The top 30 GO and KEGG enrichment results were selected and shown as bubble plots. Upregulated and downregulated genes of the KEGG pathways of interest were visualized using “Pathview” *R* package [17].

### 2.4. Evaluation of Immune Infiltration of Brain Tissues

Currently, flow cytometry is accepted as the primary way to evaluate the infiltration of immune cells. However, it may take a lot of time to excavate the potential immune cells and determine the functional phenotypes of tissues in large samples only using flow cytometry. CIBERSORT (cell-type identification by estimating relative subsets of RNA transcript) algorithm can simplify the process by deconvolution of peripheral immune cell subtypes based on linear support vector regression. CIBERSORT is one of the current methods to calculate immune cell subtypes based on gene expression profiles. In our study, we used CIBERSORT to analyze the characteristics of immune cell infiltration in brain tissues of MS. We input the merged dataset into the CIBERSORTX website (https://CIBERSORTx.stanford.edu/) and set simulation calculation times as 100 times to obtain the proportion of 22 immune cells. Then, we filtered out statistically significant data with the criterion of *p* < 0.05 for further analysis. The stacked bar chart was employed to visualize the composition of different immune cells. The correlation coefficient between different immune cells was determined and presented as a correlation heatmap. The differences between the MS group and the control group were compared using the Wilcoxon rank-sum test. The differences in immune cell infiltration between the disease group and control group were visualized by violin map using the “ggplot2” package in R. We also drew a two-dimensional PCA cluster plot to visualize the differences in immune cell infiltration between the MS group and control group using the “gggplot2” package in *R*.

### 2.5. Correlation Analysis between Hub Genes and Immune Cells

We obtained the proportion of 22 immune cells in the brain tissues of MS patients using the CIBERSORT deconvolution method in Step 4. Spearman correlation analysis was performed on the first 5 hub genes of Cytohubba and the infiltrating immune cells in the samples, which were presented as correlation maps using the “ggplot2” package in *R*.

### 2.6. Construction of Weighted Gene Coexpression Network

WGCNA [18] is an open-source *R* package that can be used to construct gene coexpression networks based on the following two points. One is that genes with similar expression patterns may be functionally related or have a coregulatory network; the other is that the connection between genes conforms to scale-free distribution [18]. In our study, we used the step-by-step method to analyze the combined gene set. Firstly, the best soft threshold *β* was calculated. Then, a hierarchical clustering tree was constructed based on the genetic correlation coefficient, and genes were assigned into different modules according to gene expression patterns. Afterward, we set the appropriate minimum quantity of gene modules and shear height threshold to further merge similar genes. The absolute value of Pearson correlation was used to measure the connectivity of genes in modules. Genes with high connectivity within the module were considered the hub genes of the module. The hub genes within a specific module often had a strong correlation with a specific trait. After obtaining the gene module of the specific trait we aimed to analyze, we used the “ggplot2” package to acquire the intersection of gene sets including MCODE Cluster 1 gene set, Cytohubba Top 10 gene set, and specific gene module from the WGCNA to observe the relationships between genes clusters.

## 3. Result

### 3.1. DEG Screening

We included GSE108000 and GSE135511 for analysis. The description of the datasets is shown in Table 1. GSE108000 dataset collected gene expression profiles of pathological specimens of brain motor cortex from 20 MS patients (specimens with or without meningeal infiltration were collected) and motor cortex specimens from 10 patients without neurological diseases. GSE135511 dataset collected gene expression profiles of 30 white matter lesions from MS patients (7 patients with chronic active lesions and 8 patients with inactive lesions) and 10 white matter samples from controls. All specimens were nonliving specimens.

**Figure 1:**
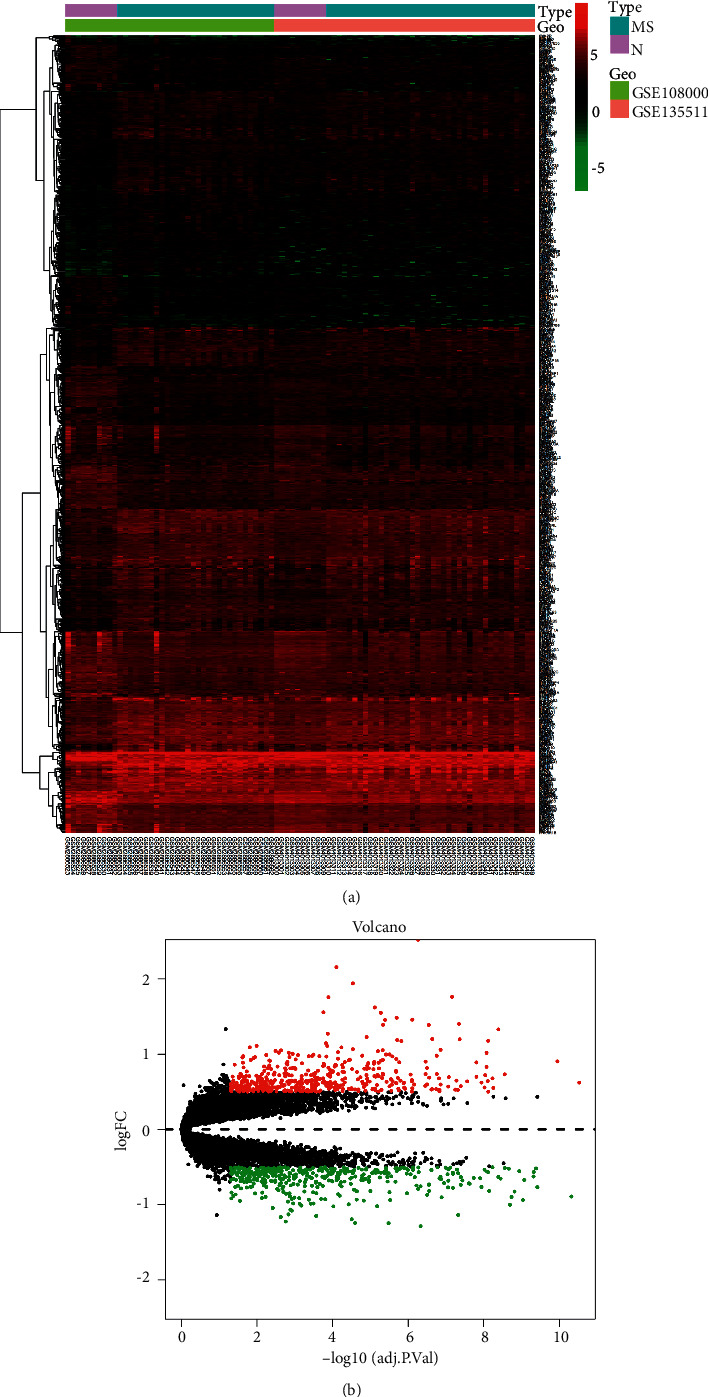
Volcano map and heat map of differentially expressed genes (DEGs). (a) The distributions of DEGs in a merged dataset of GSE108000 and GSE135511. MS refers to the multiple sclerosis group, and N refers to the control group. (b) The distribution of upregulated DEGs and downregulated DEGs in the multiple sclerosis group and control group. Color green represents downregulated DEGs and color red represents upregulated DEGs.

We downloaded the gene matrix from GSE108000 and GSE135511. GSE108000 contained gene expression profiles of white matter specimens from 30 MS patients and 10 controls. GSE135511 dataset contained gene expression data of gray matter specimens from 40 MS patients and 10 controls. We combined and normalized the two datasets, removed the batch effect between groups, and got the merged dataset. We set the threshold of significant difference of DEGs as *p* < 0.05 and | log2FC | > 0.5 to obtain more DEGs. Finally, we got 882 DEGs between the MS group and control group, including 399 upregulated genes and 483 downregulated genes (Figures 1(a), 1(b)).

### 3.2. Construction of PPI Network

**Figure 2:**
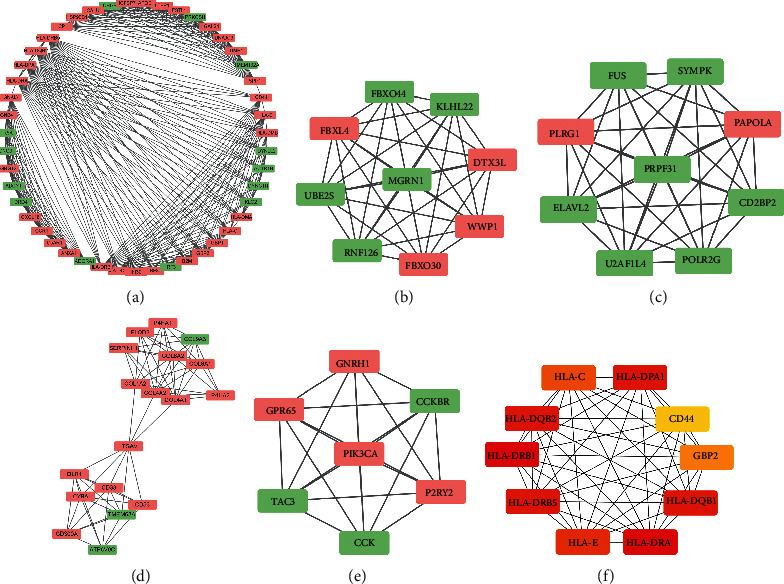
Protein-protein interaction networks of hub genes and subnetwork genes. (a)–(e) The top 5 protein-protein interaction networks obtained from the MCODE algorithm. In (a)–(e), color green represents downregulated genes and color red represents upregulated genes. The connection lines indicate the correlations between genes. (f) The interaction network of the first 10 genes obtained from the Cytohubba algorithm. The 10 genes are all upregulated genes and the color levels indicate the degree of genes in the network.

We input 882 DEGs into the STRING website, set the confidence score as 0.9, and removed the isolated genes. Then, we obtained a PPI network containing 872 nodes and 1265 edges. We downloaded network information and used Cytohubba plug-in MCC method to get the top 10 hub genes, as shown in Table 2. The genes were displayed in different color levels according to the critical degree of genes in the network, as shown in Figure 2(a). The PPI network of 872 nodes was analyzed using the MCODE plug-in, and 24 subnetworks were obtained. The first 5 subnetworks (as shown in Table 2 and Figures 2(b)–2(f)) were selected for further gene enrichment analysis.

### 3.3. Functional Enrichment Analysis of DEGs, Hub Genes, and Subnetwork Genes

**Figure 3:**
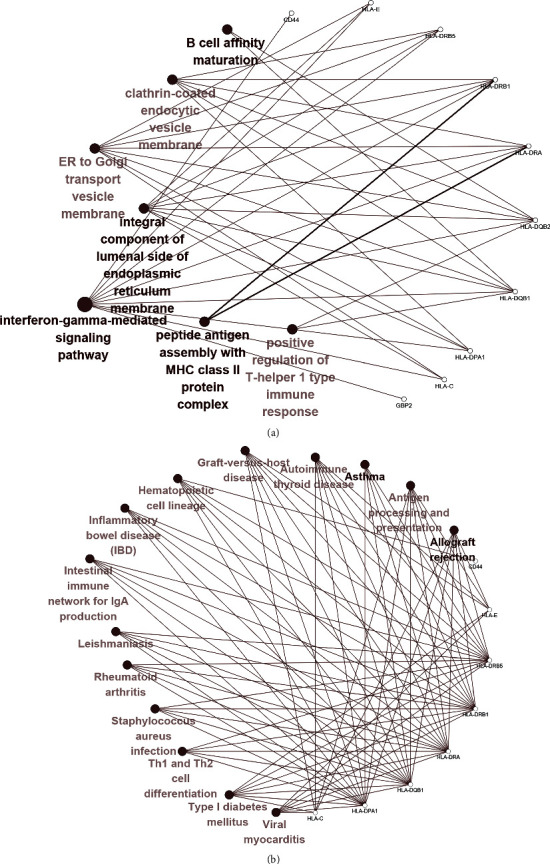
GO KEGG enrichment pathway network of the first 10 hub genes (*p* < 0.05). (a) The enrichment network of GO pathway (including BP, CC and MF) and (b) the enrichment network of KEGG pathway. The connection of the enrichment pathway suggests that there are connections between pathways and genes and between pathways. The darker the color and the larger the volume of the circle, the greater the significance of the pathway.

GO and KEGG enrichment analysis of the first 10 hub genes was conducted using Cytoscape plug-ins ClueGo and Cluepedia. As shown in Figure 3(a), the highest GO enrichment intensity suggested that the molecular functions of the 10 hub genes were closely related to the activity of MHC class II receptors, polypeptide antigens, polysaccharides binding, ER-phagosome pathway, and cytokine signal transduction in the immune system. The top 5 hub genes with the highest KEGG enrichment intensity were closely related to allograft rejection, graft-versus-host disease, type 1 diabetes, asthma, and autoimmune thyroid disease, as shown in Figure 3(b). The enrichment results of Cluster 1 obtained from MCODE were similar to those of the first 10 genes obtained from Cytohubba. GO analysis suggested that the gene set was related to MHC-II, MHC-I, glycation, growth factor binding, and amyloidosis. Cluster 2 was associated with the ubiquitin-mediated proteolysis pathway. Cluster 3 was related to spliceosome and mRNA surveillance pathway, and Cluster 4 was related to ROS and RNS production and collagen degradation by phagocytes. Cluster 5 was associated with the regulation and signal transduction of TRP pathways by inflammatory mediators.

**Figure 4:**
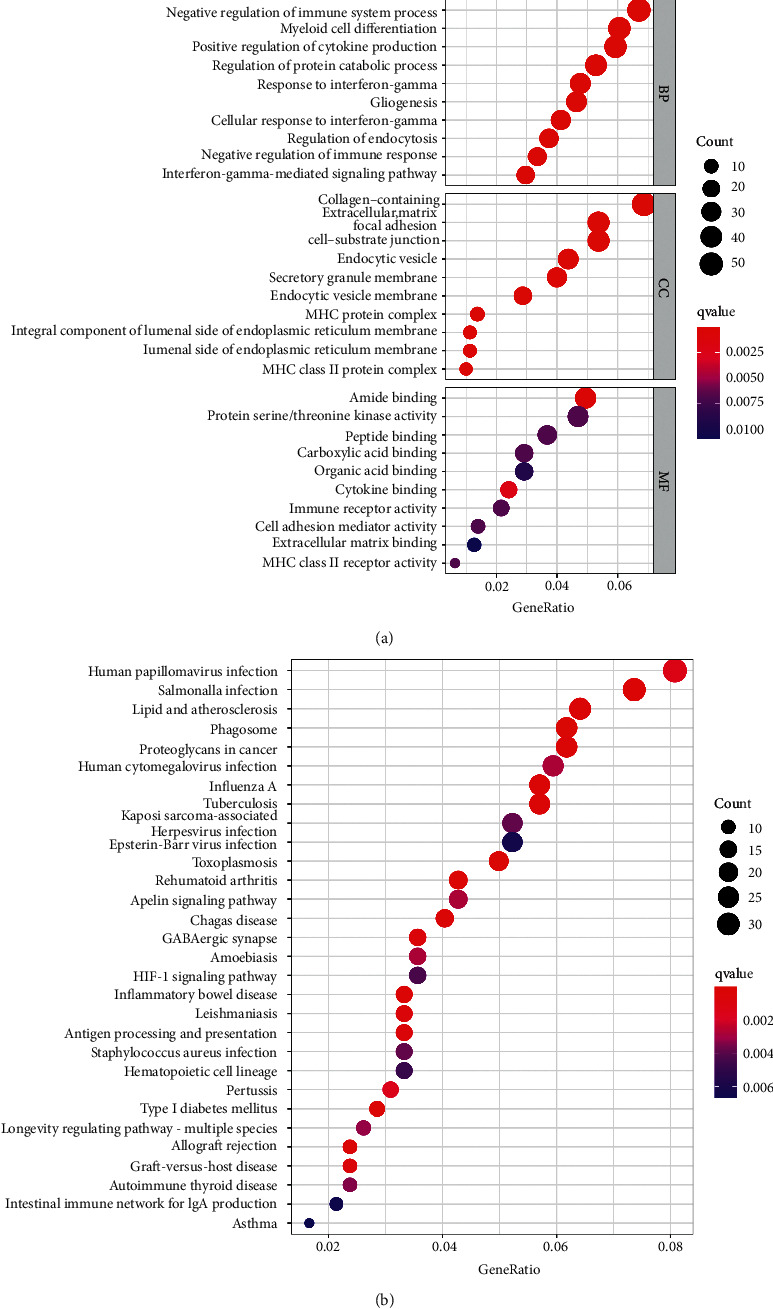
Gene Ontology (GO) and Kyoto Encyclopedia of Genes and Genomes (KEGG) analyses of DEGs. (a) GO enrichment analysis of DEGs, the top 10 enrichment pathways of biological process (BP), cellular component (CC) and molecular function (MF). Bubble size represents the number of genes related to the specific pathway. The larger the quantity of genes, the larger the bubble. And the color of the bubble represents the size of Q value. The smaller the q value, the redder the bubble. (b) The top 30 enrichment pathways in KEGG analysis. The bubble size represents the number of genes related to the pathway, and the more the number of genes, the larger the bubble. While the color of the bubble represents the size of the Q value. The smaller the q value, the redder the bubble.

GO (BP, CC, and MF) enrichment analysis was performed on the DEGs obtained in step 1 using “Clusterprofiler” in the *R* package. The most significant pathways are displayed in Figure 4(a). The most significant BPs in GO showed negative immunoregulation, myeloid differentiation, antigen and positive regulation of cytokines production, control of protein catabolism, glial cell activity, and response to the IFN*γ* pathway, etc. The CCs were mainly related to collagen fibers, extracellular matrix, adhesion plaques, endocytic vesicles, endocytic vesicle membrane, secretory granular membrane, MHC protein complex, etc. MFs were related to amide binding, protein serine/threonine kinase activity, polypeptide binding, carboxylic acid-binding, organic acid-binding, cytokine binding, immune receptor activity, cell adhesion mediator activity, and MHC class II receptor activity. The results of the KEGG enrichment analysis are shown in Figure 4(b). Enrichment pathways can be classified into virus infection (such as human papillomavirus, swine flu, cytomegalovirus, EB virus, etc.), bacterial infection (such as salmonella, *Staphylococcus aureus*, etc.), autoimmune diseases (including inflammatory bowel disease, type 1 diabetes, autoimmune thyroid disease, asthma, etc.), and others (including atherosclerosis, endogenous ligand, and other related pathways).

GO and KEGG enrichment results of the first 10 hub genes obtained by Cytoscape and Cytohubba plug-ins and the first subgene set Cluster 1 obtained by MCODE plug-in were consistent with the enrichment results of DEGs, which mainly focused on immune regulatory pathways and immune-related disease pathways. However, the enrichment results of DEGs were more comprehensive, suggesting that in addition to immune response, the response of glial cells to antigens in brain tissues also played an essential role in the pathological mechanisms.

**Figure 5:**
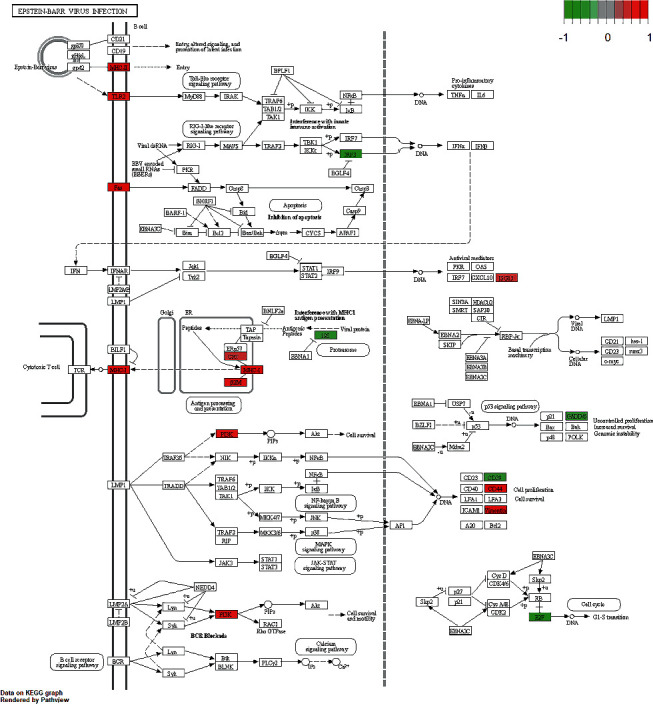
Signaling pathways associated with EB virus infection and differential gene expression in multiple sclerosis. In the figure, upregulated genes are indicated in red and downregulated genes are in green.

In our study, we also selected the Epstein-Barr virus pathway to explore the interaction between specific environmental factors and susceptibility genes of MS. The pathways were visualized using the “PathView” package in R. As shown in Figure 5, the Epstein-Barr virus mainly acted on B cells through B cell receptors such as MHC-II and TLR2. After entering B cells, Epstein-Barr virus further affected the immune activation pathway and upregulated genes related to antigen presentation of MHC-I, which further activated cytotoxic T cells. In addition, the Epstein-Barr virus might indirectly activate the PI3K pathway and affect cell proliferation and cell cycle.

### 3.4. Characteristics of Immune Infiltration in Brain Tissues of MS Patients

**Figure 6:**
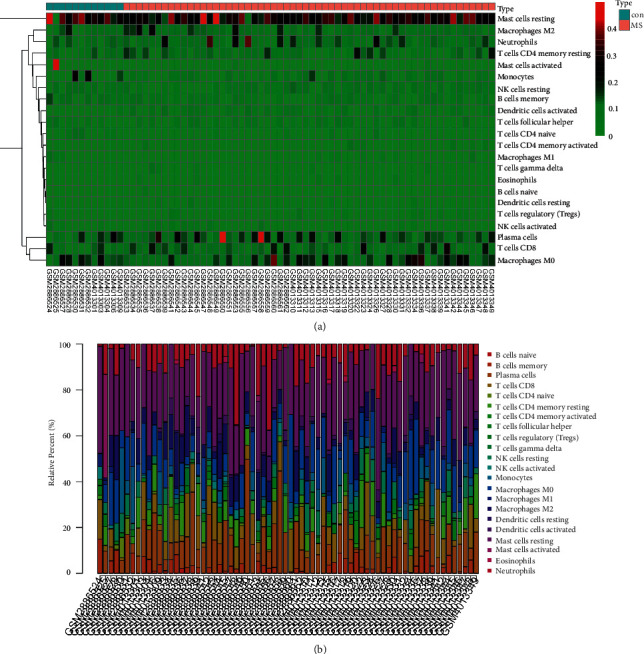
Heat map and stacking bar chart of proportions of infiltrating immune cells in the samples. (a) The expression level of immune cells in brain tissue. The darker the color, the larger the proportion. CON group represents the brain tissue samples of the control group, and the MS group represents the brain tissue samples of the multiple sclerosis group. (b) The proportion of immune cells in different tissues. The longer the bar chart, the higher the proportion of infiltrating immune cells in the tissues.

To explore the characteristics of immune infiltration in brain tissues of MS at the cellular level, we uploaded the merged dataset through the CIBERSORTX website, calculated 100 times using the CIBERSORT deconvolution method, and finally obtained the proportion of 22 immune cells in different samples. A total of 70 samples with a relatively stable proportion of immune cells (including 12 control samples and 58 MS samples) were screened out by *p* < 0.05, as shown in Figure 6. The proportion of resting mast cells, macrophage M2, neutrophils, plasma cells, T cells CD8, and macrophage M0 was relatively high in MS brain tissues.

**Figure 7:**
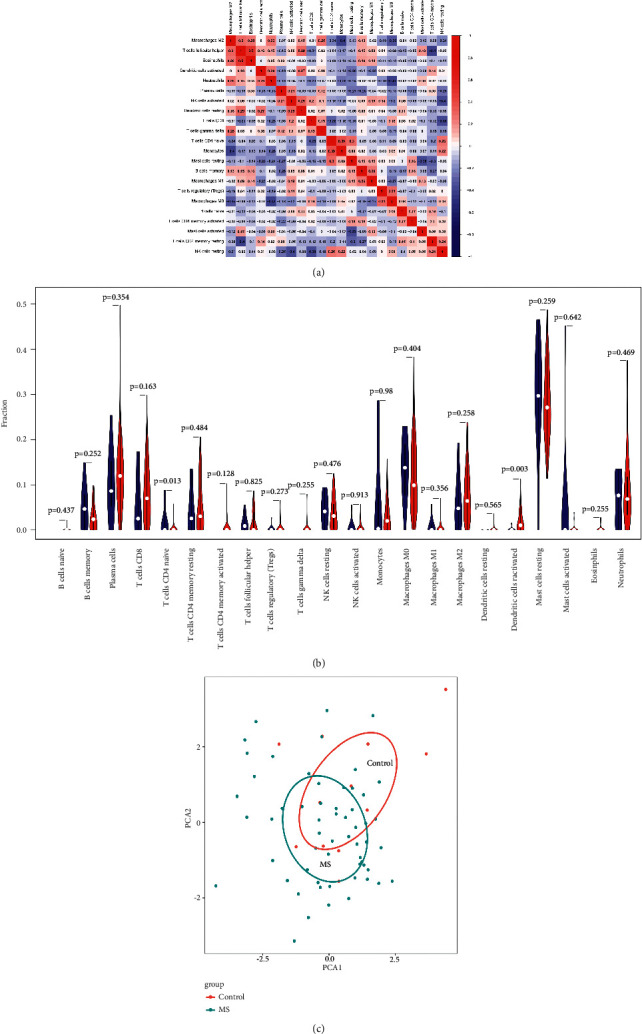
Evaluation and visualization of immune cell infiltration. (a) The correlation heat map of 22 types of immune cells. The darker the color, the stronger the correlation; color red indicates a positive correlation, while blue indicates a negative correlation. (b) The violin diagram of proportion of 22 types of immune cells between multiple sclerosis group and control group. Color red represents the multiple sclerosis group, while the color blue represents the control group. *p* < 0.05 indicates a significant difference. (c) The PCA cluster plot of infiltrating immune cells between the multiple sclerosis group and the control group.

The correlation heatmap of immune cells between the MS group and control group is shown in Figure 7(a). The correlation between follicular helper T cells and eosinophils showed a correlation coefficient with the highest absolute value (*R* = 0.5), suggesting that there was a certain positive correlation between follicular helper T cells and eosinophils. The correlation coefficient of macrophage M0 and neutrophils (*R* = −0.41) was followed, suggesting the negative correlation between macrophage M0 and neutrophils. Overall, the correlation between immune cells in the specimens was weak to moderate. The differentiation of immune cells in MS and control samples was analyzed and shown in the violin diagram. As shown in Figure 7(b), the naive CD4^+^ T cells were significantly reduced (*P* = 0.013), but the resting synaptic cells were significantly increased (*P* = 0.003) in the MS sample compared with those in the healthy control samples. The PCA cluster diagram showed that there were differences in the level of immune cell infiltration between MS and healthy control samples (Figure 7(c)).

### 3.5. Construction of Weighted Gene Coexpression Network

**Figure 8:**
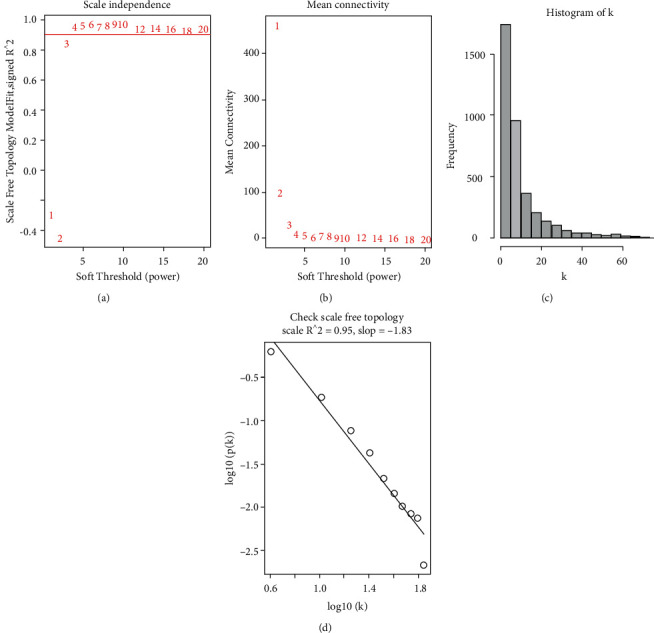
Soft threshold distribution and K value histogram. (a) shows the distribution of soft threshold. (b) The distribution of average connectivity and soft threshold. (c) The histogram of K value. (d) The graph of log10 ((p(K)) VS log10 (K), where R^2 is the correlation square between log (p(K)) and log (K). The closer the R^2 value is next to 1, the stronger the linear relationship between log (p(k)) and log (k), and the closer the constructed network to scaleless network distribution.

**Figure 9:**
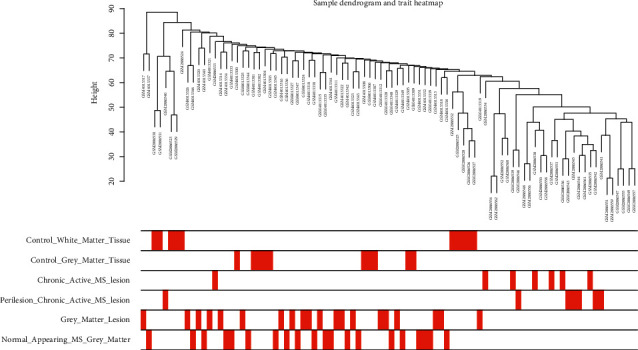
Clustering diagram of gene modules. Control_white_matter_tissue, white matter tissues of the control group; Control_gray_matter_tissue, gray matter tissues of the control group; chronic_active_MS_lesion, chronic active lesions of MS patients; perilesion_chronic_active_MS_lesion, perilesions of chronic active lesions of MS patients; gray_matter_lesion, gray matter lesions of MS patients; normal_appearing_MS_gray_matter, normal appearing gray matter of MS patients.

**Figure 10:**
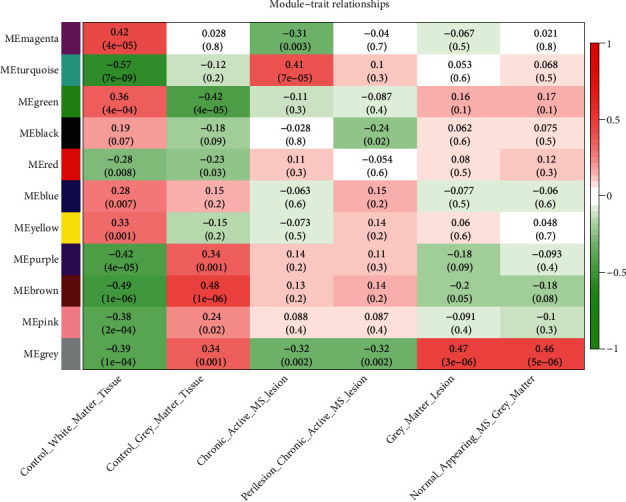
Scatterplot of correlation between genes modules and sample traits. The darker the color, the stronger the correlation between the genes module and sample trait. The color red indicates a positive correlation, while green indicates a negative correlation. Control_white_matter_tissue, white matter tissues of control group; Control_gray_matter_tissue, gray matter tissues of control group; chronic_active_MS_lesion, chronic active lesions of MS patients; perilesion_chronic_active_MS_lesion, perilesions of chronic active lesions of MS patients; gray_matter_lesion, gray matter lesions of MS patients; normal_appearing_MS_gray_matter, normal appearing gray matter of MS patients.

**Figure 11:**
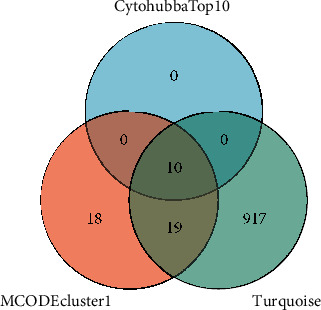
Venn diagram of intersection genes. 3 gene sets, including MCODE Cluster 1 gene set, Cytohubba Top 10 gene set, and WGCNA turquoise module genes were intersected. All top 10 genes from Cytohubba were located in the MCODE Cluster 1 gene set and the WGCNA turquoise module.

To further observe the differences in gene expression in different brain regions, we also performed WGCNA on the merged dataset to observe the differences in gene expression in white matter lesions, normal white matter tissues, gray matter lesions, and normal gray matter tissues of MS patients. The variance of all genes was extracted and the top 25% of the variance was extracted to obtain the expression profiles of 3709 genes as the input dataset of WGCNA. Based on the parameters of R2, slope, and mean *k*, the optimal soft threshold *β* value was calculated to be 4. In this study, *R*2 = 0.95, which meant that the connectivity degree of the scaling-free network construction was optimal (Figure 8). The network was constructed using the step-by-step method, and the minimum number of genes in the module was set as 30. Then, the modules with a similarity above 0.75 were merged, and finally, 11 different modules were obtained (Figure 9). We further draw the correlation heatmap between gene modules and tissue characteristics (Figure 10). Among the modules obtained, the one with the most genes and the highest correlation with traits was the turquoise module (946 genes). The correlation analysis between gene modules and tissue traits showed that there were significant differences in gene expression between the white matter of the MS group and the control group and between the gray matter of the MS group and the control group. The module with the greatest correlation with traits (turquoise module) may well distinguish the control group and disease group. There was also a certain degree of differentiation between the chronic active white matter lesions and the adjacent tissues of chronic active lesions in the disease group, but there was no effective gene module to clearly distinguish the gray matter lesions from the normal gray matter tissues. In our study, we further compared the turquoise gene module with the hub genes obtained in Step 2 and found that all the hub genes were in the turquoise module (Figure 11), suggesting a strong correlation between the hub genes and chronic active white matter lesions. There was no significant difference between normal gray matter and pathological gray matter in MS samples, but significant differences could be observed between the two gray matters and the control samples. However, in the correlation diagram between gene modules and traits, the modules with the greatest difference correlation fell in the gray module, which belonged to the unclassified gene module. In brief, compared with the control population without neurological diseases, MS patients generally had abnormal gene expression profiles in both gray matter and white matter of brain tissues. The turquoise module gene set could further distinguish chronic active lesions from the adjacent tissues of chronic active lesions in MS patients. Further information on genes belonging to eleven modules was listed in Supplementary Table S1.

### 3.6. Correlation Test between Hub Genes and Immune Cells

**Figure 12:**
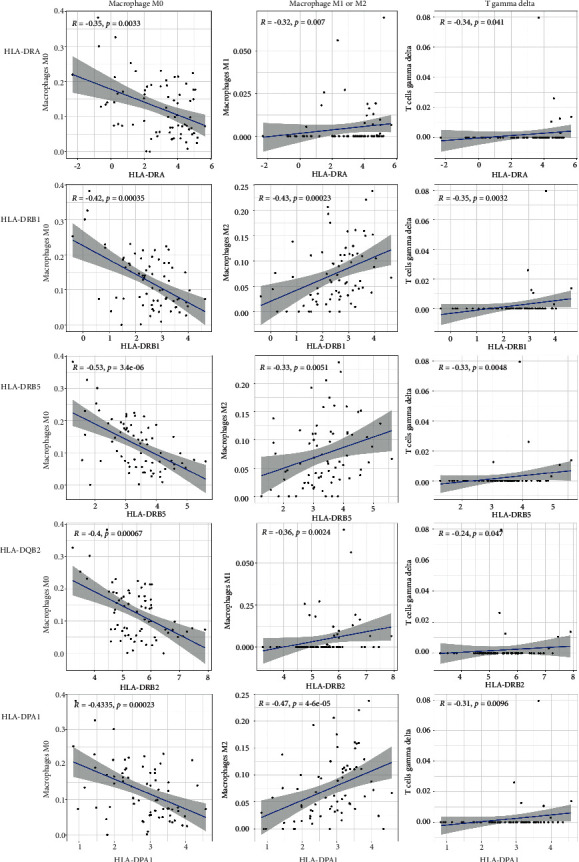
Scatterplot of correlation between top 5 hub genes (HLA-DRA, HLA-DRB1, HLA-DRA5, HLA-DRA, and HLA-DPA1) and immune cells (including macrophage and T gamma delta) (*p* < 0.05).

Spearman correlation analysis was performed between the first 5 hub genes of Cytohubba (HLA-DRA, HLA-DRB1, HLA-DRA5, HLA-DRA, and HLA-DPA1) and the 22 immune cells in the samples obtained from CIBERSORT. It was found that the expressions of the 5 hub genes were all negatively correlated with the number of M0, and positively correlated with the number of M1/M2 and the number of gamma delta in T cells. In neuroinflammatory diseases, macrophages play a dual role in the process of tissue damage according to their activation state (M1/M2). Macrophage M1 can damage neurons, while M2 macrophages are believed to facilitate the regeneration and repair of neurons, and M0 is at the resting state. The correlation diagrams of the analyzed gene correlations (*p* < 0.05) are shown as scatter plots in Figure 12.

## 4. Discussion

Multiple sclerosis is characterized by complex immune mechanisms. Specific genomic alterations drive the formation of heterogeneity in prognosis. Although there are some single genes and risk models linked to multiple sclerosis patients' prognosis, few studies were reported for analysis on the relationships of genes and immune infiltration in multiple sclerosis. The initial objective of our study was to identify the relationships between hub genes in different brain lesions and the infiltrating immune cells in brain tissues of multiple sclerosis patients.

Most hub genes identified are located in HLA-DR loci in the MHC region and are associated with immunity. The screening of the functions of hub genes also confirms that the 10 genes are susceptibility genes to MS [19–29]. However, due to the lack of previous treatment information of included patients in the original literature, we failed to determine whether the mutation is germline or influenced by treatment. WGCNA algorithm in our study also demonstrated that the 10 genes were closely related to chronic active white matter lesions. We hypothesized that the hub genes obtained in this study were significantly expressed in chronic active white matter lesions, but we could not clarify whether these genes also affected the nonlesional regions. At present, there are relatively few studies on the relationship between genes and brain regions. Some studies have evaluated the pathogenicity of NF-*κ*B signaling pathway-related genes in different brain regions of MS patients and found that genes differentially expressed in specific brain regions regulate each other [30]. The comparison of miRNAs in different brain regions of MS patients [30] has revealed that active and chronic inactive white matter lesions share regulatory miRNAs, which are mainly involved in astrocyte proliferation, microglial proliferation, and demyelination. However, microglia activation and inflammatory infiltration are not obvious in chronic inactive white matter lesions. miRNA overlap in chronic inactive white matter lesions and gray matter lesions is related to the response of astrocytes to inflammatory stimulation, maintenance of inflammatory cytokines, and apoptosis and regeneration of glial cells. Exploring gene expression in different brain tissues probably contributes to uncovering the pathological proinflammatory and anti-inflammatory mechanisms in brain tissues of MS patients.

GO analysis indicated that the hub genes were significantly enriched in immune pathways including antigen presentation, antibody production, and T cell activation, proliferation, and differentiation. GO analysis of DEGs also suggested that glial cell proliferation contributed to the pathogenesis of MS. The overall pathogenesis of MS involves peripheral immune cells and a cascade of brain glial cell activation, which is consistent with the review of Bhise [31]. Previous studies have shown that glial cells have bidirectional interactions with components of the immune system, which not only mediate tissue damage and immunity repair but also directly participate in inflammatory processes, thereby further aggravating inflammation and axon injury [32–34]. Signal transduction between inflammatory cells and target tissues may also be bidirectional. The toxic microenvironment mediated by immune cells (peripheral or central nerve cell compartmentalization) and resident cells of CNS (microglia and astrocytes) can produce inflammatory mediators, reactive oxygen species, and iron deposition, leading to chronic demyelination and axonal lesions. The structural damage of myelin oligodendrocytes and the loss of nutritional support lead to the increase of energy demand for nerve impulse transmission, while mitochondrial damage and disruption of ion homeostasis further impair normal electrical activity. The above processes jointly promote brain atrophy and explain the pathological mechanism of brain atrophy in patients with advanced MS [35, 36]. KEGG enrichment analysis in this study suggested that MS was closely related to environmental risk factors including viral infection, bacterial infection, and certain autoimmune diseases. The Epstein-Barr virus is regarded as the leading environmental risk factor [37]. The cross-reactivity of CD4^+^ T cells also partly explained the synergistic effect between EBV infection and genetic susceptibility to MS [38]. However, the pathway interaction between EB virus and DEGs of MS in our study remained to be verified. KEGG pathway analysis also suggested that MS was associated with other acquired autoimmune diseases such as inflammatory bowel disease, type 1 diabetes, autoimmune thyroid disease, and rheumatoid arthritis. MHC gene mutation is a significant contributor to the pathogenesis of most autoimmune diseases, affecting the immune cell response, T cell differentiation, cytokine secretion, and so on. Most of these autoimmune diseases are characterized by recurrent attacks and chronic progression [39].

To further explore the role of immune cells in the brain tissues of MS, we used the CIBERSORTX algorithm to analyze the brain tissue samples from MS patients. In general, the number of CD4^+^ T cells in MS samples is more than that in control samples. Our results showed a decrease in initial CD4^+^ T cells, suggesting that the proportion of CD4^+^ T cells in brain tissues of MS patients may vary with cell subtypes [40]. CD8^+^ T lymphocytes are resident cells of the brain and spinal cord tissues and transmit neuroinflammation locally when the cells encounter homologous antigens [41]. MS has long been viewed as a disease mediated by T cells, but in recent years, accumulating studies have focused on other important components including B cells, endothelial cells, complements, autoantibodies, cytokines, and chemokines [42]. B cells and myeloid cells are equally important in the inflammatory response of CNS. At present, anti-CD20 monoclonal antibodies (such as rituximab) are clinically used to deplete B cells, which can reduce the recurrence and retard the progression of the disease, further verifying the important roles of B cells and antibodies in MS progression [43]. Macrophages and microglia have similar inflammatory effects, and the infiltration of macrophages is strongly correlated with the model of advanced EAE [44], suggesting the role of macrophages in the advanced stage of the disease. EAE-related studies have found that the inactivation of macrophages can improve the severity of the disease [45]. Permanent myeloid cells can be found in all tissues and organs. In the case of infection or tissue injury, monocytes or granulocytes (especially neutrophils) can be recruited from the circulation to participate in the promotion of inflammatory response at the beginning and play the roles in tissue repair and regeneration at the later stage [46, 47]. The previous study on immune cell patterns of gene expression has suggested that the development of MS is caused by a wide subset of cells [42]. Patients may have a dysregulated proportion of immune cells, which results in differences in treatment responses. Susceptibility genes for major cell subsets are responsible for transcription factors, chemokines, receptors, intracellular enzymes, and signal transduction factors that control cell proportion, cell differentiation, and cell status [42]. Identifying the target antigens by tissue-hosting CD8^+^ T cells and B cells and recognizing the molecular properties and corresponding gene targets of soluble inflammatory mediators that may cause tissue damage are conducive to the treatment of MS [41]. Some hub genes were found negatively correlated with immune cells at the resting state and positively correlated with immune cells at the active state, suggesting that certain hub genes were related to the activation of peripheral immune cells such as macrophages. However, the role of genes in the regulation of immune cells in MS patients remained to be further investigated.

The 2019 International Multiple Sclerosis Genetics Consortium (IMSGC) explores the expression of MS genomewide immune cells and immune organs [8]. IMSGC annotates genomewide gene fragments of interest using gene expression profiles and epigenetic signature information and demonstrates that MS susceptibility loci are significantly enriched in many different immune cell types and tissues, including natural killer cells, dendritic cells, and thymus. Different from the IMSGC study, our study intuitively shows the changes of the pathological microenvironment of brain tissues.

However, our study has the following limitations, similar to most bioinformatics studies. Firstly, the sample size and patient information are limited, and we can not eliminate the bias of gender, comorbidity, disease stage, and medications. Secondly, although the merged data increase the sample size, the systematic error caused by different research methods and experimental conditions still cannot be eliminated. Thirdly, the samples in this study are from cadavers, and the gene expression profiles of these samples may be affected by the sample storage time and processing methods, which are somewhat different from living tissues. Although most of the results obtained in our study are consistent with previous studies, more studies are needed for further verification.

Given the fact that MS is not a simple genetic disease, and there are complex interactions among genetics, environment, and immunity, it is difficult to realize the application of genes in the clinical diagnosis and treatment of MS. Currently, more and more studies focus on the interaction between genes and the environment and the underlying immune mechanism. However, future studies should consider more information including clinical phenotypes, prognosis and treatment response, genetic (including epigenetic), and pathology characteristics. Moreover, it is necessary to expand the sample size to further explore the molecular mechanism of clinical phenotype and therapeutic prognosis heterogeneity, which will contribute to further clarifying the molecular classifications of the disease and guiding disease prevention and medication development of MS.

## 5. Conclusion

In this study, 10 key genes of MS including HLA-DRA, HLA-DRB1, HLA-DRB5, HLA-DQB2, HLA-DPA1, HLA-DQB1, HLA-E, HLA-C, GBP2, and CD44 are obtained using the algorithm. Enrichment pathways are associated with T cell activation, proliferation and differentiation, antigen presentation, autoantibody production, and glial cell proliferation, suggesting that the pathogenesis of MS was correlated with the involvement of the peripheral immune system and a cascade of brain glial cell activation. The infiltrating immune cells in brain tissues are diverse and are not only related to T cells but also B cells, macrophages, granulocytes, and other peripheral immune cells. The key genes are correlated with some immune cells, indicating the role of susceptibility genes in promoting the activation of macrophages and natural killer cells. The gene expression profiles in MS brain tissues (including gray matter and white matter) are significantly different from those in the control samples, with the chronic active white matter lesions showing the most significant differences, followed by the tissues adjacent to chronic active white matter lesions. The 10 key genes are closely related to chronic active white matter lesions. The analysis of DEG pathway enrichment and peripheral immune cell infiltration can confer novel insights into the etiological and pathological mechanisms of MS. The gene expression profiles and underlying mechanisms of immune infiltration in MS and their correlations with environment and disease stage need further exploration.

## Figures and Tables

**Table 1:** Basic information of data from GSE108000 and GSE135511.

Dataset ID	Sample source	Data platform	Sample grouping	Disease subtypes	Sample number	Published date
GSE108000	Brain tissue	GPL13497 (agilent)	Multiple sclerosis and control	SPMS	40 (7M/18F)	Jan-18
GSE135511	Brain tissue	GPL6883 (illumina)	Multiple sclerosis and control	No specific	50 (14F/16M)	Dec-19

SPMS, secondary progressive multiple sclerosis; M, male; F, female.

**Table 2:** The first 10 core genes obtained from Cytohubba plug-in and genes of 5 gene sets from the MCODE plug-in.

Gene sets	Gene lists
Top 10 hub genes	HLA-DRA, HLA-DRB1, HLA-DRB5, HLA-DQB2, HLA-DPA1, HLA-DQB1, HLA-E, HLA-C, GBP2, CD44

Cluster 1	ACTR1B, HLA-DRB5, HLA-DQB2, HLA-DPA1, HLA-DMB, B2M, HLA-DRA, HLA-DMA, HLA-DRB1, HLA-DQB1, IRF8, KLC2, DYNLL2, DYNC1I1, GBP2, GBP1, DNAJC3, HLA-C, HLA-E, IFI30, CD44, IRF3, TMEM132A, LGALS1, PRKCSH, FSTL1, LTBP1, IGFBP7, CHGB, ADCY1, CALU, DRD4, HSP90B1, GNB4, HTR5A, CXCL16, CP, GNG3, CCR1, SPP1, C5AR1, ANXA1, GNG12, ADORA1, GNAI3, APOE, TIMP1

Cluster 2	WWP1, FBXO30, KLHL22, FBXO44, FBXL4, UBE2S, DTX3L, MGRN1, RNF126

Cluster 3	CD2BP2, PAPOLA, SYMPK, FUS, U2AF1L4, POLR2G, ELAVL2, PLRG1, PRPF31

Cluster 4	OLR1, CD300A, TMEM63A, ITGAV, COL8A1, ATP6V0C, P4HA1, PLOD2, COL1A2, COL9A3, COL8A2, P4HA2, COL4A2, CYBA, COL4A1, CD33, SERPINH1, CD53

Cluster 5	GPR65, GNRH1, P2RY2, CCKBR, TAC3, CCK, PIK3CA

## Data Availability

The datasets analyzed during the current study are available in the National Center for Biotechnology Information-Gene Expression Omnibus (NCBI-GEO) database (https://www.ncbi.nlm.nih.gov/geo/). We downloaded two datasets: GSE108000 and GSE135511.

## Supplementary Materials

Supplementary Materials Supplement Table S1. Coexpressed genes of the eleven modules.
