# Supplementary material for: Integration of Genetic and Immune Infiltration Insights into Data Mining of Multiple Sclerosis Pathogenesis
Source: Comput Intell Neurosci. 2022 Jun 27;2022:1661334. doi: 10.1155/2022/1661334 (PMC9252675; doi:10.1155/2022/1661334)
Supplement: Supplementary Materials — Supplement Table S1. Coexpressed genes of the eleven modules. [file 1661334.f1.docx]

Supplement Table S1.Co-expressed genes of the eleven modules

| Module | Gene |
| --- | --- |
| black | OTUD7A, LTBR, TAPBP, ARHGEF16, ZGPAT, ADH1A, CACNG1, GRP, OXGR1, ALAS2, DUSP10, ACTA2, FCN3, KCNK12, TRIM23, C6orf191, NAIP, SCRN3, CRISPLD2, CA1, SEC14L4, MAGEB10, ANXA3, PARP10, ITGA8, TNFRSF6B, PBX4, ABCG2, GJA4, IER2, PRSS35, KLRC1, MCCC2, VTI1B, ST3GAL4, GIP, SLC22A7, PACSIN3, C16orf78, CYP24A1, ATP4A, CCL19, RTN4IP1, TBX1, CFB, APOL3, PLA1A, ARHGAP29, CXCL1, CLIC3, SELE, GRB14, C19orf34, FOXC1, SELT, GPR4, ETV1, IL2RA, ZMYND12, SLC2A3, USP14, MAMDC4, AOC3, TBX2, LHB, PPARD, MMP28, GDF1, IGFBP3, LRRC32, C17orf78, CCL16, GADD45G, BMP4, ZNF396, CAPS2, CST9L, EFHB, CNN1, TIE1, BMP6, CTR9, C19orf33, KRT5, SLC16A12, PEPD, EN2, HAPLN3, HCRTR1, SLC4A4, CD8B, TTC7A, CLCA1, IL18R1, NTF3, NPTX2, ALPL, ADAMTS1, GRRP1, C1orf64, SLCO4A1, FMO1, KRT19, BCL3, MYNN, HGD, ADCY4, ANGPT2, EVX1, PKIA, MYO9B, FTHL17, SOCS1, CFHR3, LUZP2, MLXIP, S100A5, CFH, C15orf42, PRKRIR, APOLD1, GDF11, APOH, OVOS2, BARHL2, IGFBP2, HIF3A, ENG, C20orf54, TNFRSF4, FHL5, NOSTRIN, HSD17B8, PFN4, GP9, KRTAP10-4, MMP3, TBXA2R, ACVRL1, C1GALT1C1, OSTN, CDC42, WNT6, PAPSS2, LCE2A, PKN3, KRTAP26-1, CLDN5, ABCB1, DGKD, EPS8L2, PLCH1, S100A7L2, ACTG2, GJA3, A4GALT, CCHCR1, CEACAM5, ATOH8, S100A1, IL9R |
| blue | DPEP2, FGR, SLC37A2, CCL5, MFNG, PLXDC2, PLCG2, HS3ST2, IRF5, DYNC1I1, IL7R, TCIRG1, CIITA, C20orf141, OR5D13, APBB1, LRRC8B, SLC36A1, PREPL, ICAM1, ACOT7, NBPF14, VASP, CYP3A4, SLC6A16, KCTD8, SLC16A3, TBC1D16, MS4A1, PLEKHF2, GLP1R, MYO1D, GALNTL2, FAM107A, CYP4F12, SERINC3, EFHA2, DIRC1, TSGA10, ANXA13, RAB35, NIF3L1, ITGA7, SAE1, FAM107B, LGR5, GRIN3A, UGT8, COL3A1, ECM2, RN7SK, ARHGDIG, UGT2B7, OR11L1, SERPINA12, SETDB2, CCR9, LTB, PHF11, OGDHL, TRPC1, SMPD1, ABCA6, CGNL1, NET1, PUS1, CNTNAP2, GJB2, DRD1, PTPN18, EMILIN2, CD99, SLC25A17, HOXC4, PCP4, RELN, SRGAP2, ADH1C, ZNF445, CHRNA10, PAFAH1B3, APOBEC3D, ASB5, MYO5B, UNC13C, SSFA2, EGR1, GPR6, LY6E, TUBA8, TALDO1, KLF2, PSD4, FOXA2, CFTR, PRSS3, IL16, KCNJ10, FLRT3, TMC8, PLK3, SPN, C21orf88, PRRT1, ZNF540, PTPN13, OR7E24, NBPF3, LCTL, CD3D, MITF, MAP1LC3A, TAC1, PNLIPRP2, TNKS, PDXP, ZNF621, PDE7A, FAM9B, UGT2B11, POU3F2, ACTR3B, PPARGC1B, SELK, PLA2G3, CYP4F8, IL27RA, CPNE4, ST6GAL2, DAO, UGT2A1, MFN1, TMTC2, AGXT2, ADCY1, CXCL3, CRYM, C15orf5, KRTCAP2, C8orf22, COX7B2, GALE, GFER, FANCL, SIX5, PCDHA1, GLRA2, FUT8, GREM2, FNTA, COX7A1, BID, KRTAP17-1, NRN1, FLJ16124, AMOTL2, PAPLN, PNRC1, SERPINA4, LCN12, STON1, REEP3, TEKT2, TAGLN3, LRFN2, ADCYAP1, FRZB, CDCA7L, C1orf158, PAK7, PAX1, RAD51AP1, LIPC, BRMS1L, ANGPTL3, CHRDL1, BCL2, PITPNM2, C6orf118, CDH12, LOC441268, SPHAR, IL1R1, FMO3, ZNF300, HIVEP3, DEPDC1, NFATC2, CDK5R1, UBE2R2, FSTL4, POPDC3, IRX2, SLC25A11, SYNGR3, WTAP, HOXA7, TAF5, DLGAP3, ADRB1, SLC25A22, SIDT2, CBLN4, ANLN, CXCL9, SERF1A, CRIP1, SPARC, CALB1, C9orf4, DIRAS2, PCDH7, C8orf47, HIST2H2AC, FGF7, PAPOLA, CYP1B1, HSP90B1, HOXA3, GCGR, KLHDC7A, MCOLN3, CPLX2, CABP1, CAMKK2, ZNF451, ACSL5, HIST1H1E, RHBDF1, EGR4, STAR, CISH, SLC8A1, SLC22A15, PPM1E, NUDCD3, VSX1, ATRNL1, KCND2, KRTAP4-5, COPS8, GNB5, KIF17, SLC10A1, CXCL14, IL32, LOC650293, TAC3, ARRDC4, EFNA1, INA, COL5A2, ATP2B2, DPP6, ZCCHC13, ESCO2, HTR5A, C1orf110, SLC22A17, COL1A2, OR2A20P, TACR3, IL12A, TUB, CAMK2A, SLC39A10, HPX, SLITRK5, HYAL1, CAV1, ZDHHC20, DEFB1, MAP7D2, OR7A5, NCDN, CCK, HERC2P4, CYP39A1, ALS2CR11, HIST1H2AE, CREB3L4, BTN1A1, TEF, ELMOD1, HAO2, ANKH, MC1R, BTBD8, CLIC6, OR13A1, BEX1, KLRC4, CAPS, GLS2, TBL1Y, SERPINA7, BAIAP3, RAC3, KRTAP5-8, MEF2B, FAM19A2, C13orf16, OR10T2, PSMA2, HOXD8, TMCC3, PRPF31, BASP1, TEAD2, BLK, ANXA10, RAB23, OR10V1, SLC9A9, APOA5, PELI1, NBPF1, TCERG1L, SH2D3A, GABRG2, ICAM5, APOD, ATP1A3, ACOT6, TGM1, SLC27A6, CORO2A, APLN, EGFL6, KRT18, PRKG1, SLC1A1, IRF3, MS4A2, CRYAB, OR52A1, MMD, PROC, C21orf49, SLC30A3, CNGB3, TAF6, MAP3K7, APOB, ZNF639, TTYH3, CDH8, FLJ42102, MAP6, ZNF354B, OR6Q1, NEK1, C6orf168, C12orf36, ZNF192, IL7, PLEKHJ1, LHX5, GABPA, OR8B8, IL4R, FOXD2, KIAA1432, OR51E2, PPP1R1B, RAB3C, C1orf173, RBMS3, SENP7, KCNC1, MAL2, ARL4C, AFM, FGF13, CBLN2, PPP1R1C, NEURL, EPO, PDZD11, TTTY10, NGB, STAU1, OR8D2, CYP3A7, C4BPB, TMC6, RASSF1, GPR45, IGF1R, CES1, PNPLA8, OR8I2, SPRN, SMC2, LAMP1, CSMD3, ZNF710, PRKAR1B, PAPSS1, C3orf20, S100A9, C12orf11, CPNE7, PTPRN, MAGEA8, C10orf91, TNKS2, ISLR, KCTD2, EDN1, FBXW10, TP53INP1, TFDP2, PRODH2, HOXB3, KCMF1, TCEAL2, STARD13, ATM, TTLL5, DIRAS1, THAP5, RN7SL1, MMP24, FIP1L1, GABPB2, C6, SATB2, OR6C3, CLEC2B, SLC39A5, LOC388692, NNAT, SLC17A6, STAC2, HAPLN4, VGLL2, WDR78, IFNAR2, TESK2, ZNF207, RYK, SNAP29, NPNT, TCF12, ATP13A4, FBXO25, PLXDC1, KLF16, MAGEF1, CAMK1D, KRTAP5-11, CD1B, LYPD1, GSC, D4S234E, ZNF480, CYLC1, CDH13, CTGF, CAMK4, LBR, AKR1B10, PCYOX1, GRIN2B, C20orf112, CCKBR, APBA1, FLNC, CHGB, FAT4, CCDC36, WBSCR17, SYNCRIP, ARGFXP2, RB1CC1, KCNA5, MYOM2, FBXL4, TAF1, GSTA5, TFDP1, PAX5, KCNT1, RBM25, SLC4A8, PHKA2, SKI, RPS26, NPHP1, GLRA3, KRTAP13-3, SELS, CKMT1A, EPDR1, EXTL1, TNRC6B, NKRF, PITPNA, MINA, TPSD1, SOX8, SSTR2, EPN3, C9orf117, ACTA1, FUT10, MYLK, MCEE, PRICKLE2, PADI2, SNX1, ANKRD5, SLC4A10, KCNA4, IL21R, STX2, CXCL13, CPAMD8, FOXD1, COL18A1, OMA1, CRP, AKAP7, DCN, NAV1, OR51D1, GABRA2, CA5A, OR2L13, NRXN1, PLXNA3, CPO, IRX1, CD5L, HIST2H2AB, APOA4, CCL7, SMYD3, OR10AD1, RNF13, SLC25A37, FBXO40, CYP2W1, FGF12, SCN1A, HOXB13, AMPH, HECW1, KLC2, INHBC, CTXN3, FBLN2, LCORL, NTS, HIST1H2BM, NDFIP1, TRIM54, NDUFB10, SPAG8, CRYGB, KCNA1, SCYL3, SLC26A2, FHL2, SORBS2, RTN4R, GLT1D1, B3GNT7, KCNS1, FBXL16, PROP1, HPCA, KCNJ6, SGCA, GAD2, ZNF223, CRLF1, OR52J3, LOC389834, KCNB2, ARL6IP6, LRRC42, RPUSD3, GPR123, SLC16A14, NPTN, POSTN, APOBEC4, ESM1, FBXW7, MAGEB18, UNC13A, ZNF549, FABP1, GABRA1, FOXE1, CLDN3, DNASE1L3, ANGPT4, KLKB1, CPN1, CCR8, C8B, ULBP1, PRRT2, DENND2C, CHRNB2, CHST6, OR6X1, ENC1, TPSG1, UBE2NL, OR9G4, APOF, NMT1, C3orf52, CAMK2B, DUXA, PHYHIP, FAM120C, P2RY1, KIN, SNAPC4, TEX14, CXCL11, FRMPD2, CNR1, CYP1A2, NLGN4X, GABBR2, QSER1, ASPN, FKBP7, VAV2, LOC643923, FAM46D, CNIH3, PACSIN1, SAGE1, WNT2B, GPR68, CXorf22, MAGED2, ZMYND19, GABRA5, SULT4A1, SH3BGR, PPFIBP2, FGF14, NIPSNAP3B, DLGAP1, GZMM, DUX4, NKTR, SNTG2, ITLN2, FMO2, SSX2IP, LRRC2, SLITRK6, PDZD3, FDXR, CNTF, GSTM5, CLSPN, CPNE5, REEP4, HAPLN1, HRK, UGP2, NEDD8, OR8H1, CLLU1OS, EPHA5, NOVA2, ZADH2, CRH, C9orf85, PANX2, RAD54B, HHLA1, BAGE4, GNG2, HPCAL4, DACT2, SAMD14, CACNB4, GPM6A, IGF2, FUT9, NEUROD2, WIPF1, C7, CNOT1, C1QL4, MEGF8, ELAVL4, CLEC1B, OR4K14, TAS2R46, ITM2A, CHRM1, PKD1, SLC10A5, BCL11B, PRKAR2B, KCNQ3, IL24, ZP4, OR6C65, IGHMBP2, HAL, ATXN7, DKK3, GABRB2, DGKZ, CADPS, GSTT1, ABCB4, IL8, TCHP, PAFAH2, SORD, ADAM20, C20orf103, GSTA2, INHBE, DDX59, EPHB6, SERINC5, LPPR4, HIST1H2BB, TJP2, IL6ST, OR5B12, SLC6A17, CCL20, FRMPD4, SST, 1-Dec, DCDC1, PNOC, CCDC42, GDA, ARNTL2, TTN, TTC25, GOLGA8E, IQGAP1, MRGPRD, SLC26A1, CALD1, OR51A7, FMOD, GPR26, OPA3, RIN1, SYT13, LGI2, SLC35B4, CHGA, TMEM89, NR1H2, SNURF, HAVCR1, CPNE9, HYAL3, NDRG4, SLC38A5, COL1A1, TUBGCP3, HEY2, TTLL9, KCTD10, INS-IGF2, SCN9A, SCN3B, DUSP5P, HTATIP2, SALL3, OR2G3, OR51I2, ENTPD2, OR10G9, MAN1A2, IFRD2, THYN1, THRSP, MBD3L1, TFE3, SOHLH2, SCN5A, OPN4, BTBD11, AQP3, ANP32D, SLC5A10, CAMK1G, C10orf71, OR5D14, KCNB1, SLC23A3, VIPR2, ADRA1B, CCDC37, KLRC3, NAT10, GJB7, BMPER, C1orf127, MED19, EDARADD, KIAA1033, FBXL3, CPLX1, MORC2, MLANA, GPR52, OGFOD2, HIST1H2BK, ZNF132, FAM3C, SKIV2L, PAGE2B, DPP10, CREG2, PYCRL, TIMP4, SYNPR, TRIM28, CLEC3B, HIST1H2AC, EPHA4, ULK1, TRO, CENPI, KCNH5, ZNF134, ZNF141, NPTX1, H2AFJ, PRSS27, C1QL3, HIST1H2AD, NGEF, MGEA5, NR2F6, SLC12A8, CLSTN3, PSG2, GRIA1, CXCL2, PQLC3, LBP, LATS1, TDRD7, RPS6, GATA2, OR56A3, CAP2, KRTAP19-1, TAS2R45, CCL15, RTF1, ST3GAL5, CHRND, OR11G2, CCL22, TMEM2, CIT, CRYBA1, CACNB2, ATP2B1, ZNF582, CDH18, TMEM185A, GDF6, NLGN2, RNF20, CPEB3, GLP2R, MYH14, C1QTNF4, CYP2A7, LCN2, CYP2B6, SLC45A1, GAP43, ZNF358, TUBB3, MCTS1, CHD3, NOL4, C13orf26, HIST1H2BD, SOX11, ELAVL2, CDH19, SPARCL1, CHN1, HCN1, IL1RN, CAMK2G, TAS2R38, NUP160, ATP13A5, TMEM26, ASNA1, CBFB, FKBP1B, FAT2, IL12RB2, EPHA7, CRYBB3, PRUNE2, GALR1, SLTM, XRCC4, EGLN2, PCDHGA3, IRX4 |
| brown | S100A4, CD83, SULT1C2, NCF2, MFSD1, SGSH, RGS19, RAB11FIP1, MOBP, HSPB2, TNFRSF12A, TRIM36, SYNJ2, TGM6, MDFI, HIGD1A, GPR171, PRRX1, FSTL5, MEI1, HINT3, ABTB2, TSPAN8, KRTAP5-6, SGK2, IDI1, CHKB, TSPAN14, SLC16A8, MAGEB3, GNRHR, THOC4, TRIM5, C14orf37, OR51V1, OR1M1, SLC12A7, USH1C, MYF5, VEGFB, LRRC37B, GLIPR1, LOC93622, C9orf152, ACAT2, KAL1, GLYCAM1, ADH4, DIO1, ITCH, EPB42, LOC392196, PTGS1, IMPAD1, LCE3B, DUOX2, SLC12A1, SLC30A10, FUCA2, CACNA1S, WDR45L, UGT2A3, OR52D1, DEFA3, KRTAP4-2, OR8G1, SSR2, NUDT2, LCN6, FOXA3, OR1A2, APOL1, HSPA12A, MBTPS1, CD300LB, CASKIN2, KRT39, SORBS1, KRT10, TM2D3, MUC1, MMACHC, C20orf106, CCR4, SLC6A19, LCE1A, FGFBP1, SERPINB10, OR2D2, MYH11, ADAM3A, PNMA3, OR2V2, FNDC7, MAP2K3, OR5A2, DOCK4, IMPDH2, C6orf201, LCN10, SYNPO2L, TMOD4, STK38L, OR8K5, CLDN10, SLC30A9, FSHB, GCC2, ADAMTS12, PTPN12, FRK, CD24, ZNF655, OR56B4, C6orf10, OSTF1, MRRF, SLAMF6, RGS22, PLAC8L1, OR1L3, PCDHB16, EEF1E1, FCN1, C10orf113, OR2W3, ANP32C, SNX4, GABRR1, GNPDA2, SUCLG1, ZSWIM4, IL29, PAH, CTRB1, CALML5, MORC3, OR6K2, LY6K, 5-Mar, OR1C1, KIR2DL4, MUM1, HOXC8, EBF2, USP50, FAM71C, RNF170, DOK5, HRC, NEDD4, CHAC2, GPR110, KLF8, USP38, LRRCC1, PSMF1, OR51M1, OR2F1, KRT1, OR2H1, COL24A1, PDSS2, KLK10, SLC25A15, SDHB, ENTPD7, SPACA3, WWP2, MLC1, OR6C1, HSF5, CETN1, SNX11, OR4K1, OR10AG1, TTTY19, OR10A3, ART5, NCBP1, POU5F1, ADAMTS3, TP73, GALP, KIAA1024, SFMBT1, CSH1, SACS, USHBP1, MIS12, PSMA3, HDC, ALX4, FLJ25328, C12orf42, RASGRP3, TPH1, DEGS2, GPRC5D, C11orf52, MESDC2, CEACAM8, EIF4G1, VENTX, MPHOSPH6, PAGE1, GTF3C4, GATA4, KRT12, MIF4GD, C20orf152, WFDC11, ZNF660, DCHS2, C18orf25, TTLL7, MAOA, PELI2, ARFGAP3, FABP4, TMPRSS11D, OR5H1, OR51G1, CITED1, OPLAH, NCOR1, POU3F3, OR4D11, DTWD1, FLJ45831, LRRC55, TRPV4, CHID1, TREX1, SLC30A1, CHMP4C, OR2Y1, T, FOXL2, BHMT, TTTY2, FCRL6, KIF23, SLC5A1, C15orf43, FRMD1, FATE1, ASB6, ELF3, SGPL1, OR2AK2, DLEU7, SLC10A7, FAM126A, TAAR2, TAS2R16, RPAP1, KIFC1, ODF2, PCDHAC1, ATRX, SLCO2A1, TTTY1, NEUROD4, SERPINB11, SLC39A3, NEIL3, OIP5, OR8J3, PIGY, XAGE5, LGR4, OR6Y1, SF3B3, SSR3, SDK2, SPAG16, RBMX, PRKRIP1, CDC42BPG, UBQLN3, KRT86, GBP7, GRIP1, SULT1A4, SULT6B1, NEB, KIR2DL5A, KRTAP10-2, OR5P2, FCRL4, C15orf23, KIAA1530, CSRP3, NKX6-1, CENPE, OR1D2, LCE1D, CYP2F1, TTTY5, CTAGE1, C3orf24, SLC25A34, VPS53, C10orf129, BAAT, CYP4Z2P, FGF22, C12orf40, TMPRSS11A, SSSCA1, GAL, HEPH, C20orf134, BMP10, TSGA13, ANKRD56, PI16, CCDC65, LRRC37A2, FTMT, NEURL2, OR8U1, PQLC2, ARSE, SCD5, NOLC1, KRT4, CASR, RASSF6, AP1G2, PTN, JAK3, OR6N1, SLC25A26, PRKACG, TEX11, NEU4, PCDHB8, MYL5, YARS, SCGB1C1, TDP1, PPP6C, CALML6, OR2T27, FAM47B, PEBP4, OR2T12, LRRC31, NEIL1, STC1, CCDC108, NRIP2, PDZD8, C6orf146, IGFL1, KREMEN2, OR2A42, PHOSPHO2, OR4C12, HOXD10, FAM54A, CSN1S1, SURF2, TRIM32, EPAG, OR4F21, TREH, GULP1, CILP2, KRT23, CLEC12B, WDR64, PPP2R5E, NGLY1, DPP8, KRT26, NMNAT3, BIK, TMOD1, RGS3, SCARA5, NPLOC4, RTCD1, SERF2, TNMD, DPCR1, TLL1, AREG, CHRDL2, MC3R, SULF2, GOLT1A, DCDC2, RASA1, GAB2, PCDHB11, ESPNL, CEACAM20, ACBD7, F2RL2, SERPINC1, C20orf195, OR4F6, MECR, GRID2, KRTAP27-1, FBXO47, ZNF100, OR5AN1, EPX, OR6C4, IFNW1, OSTalpha, WBP5, DTYMK, ZC3H8, HTR2B, WHSC1L1, GADL1, DAB1, CHM, SYT8, TEX264, GDAP1, CCDC129, CDKN3, MBD5, FAM55A, CRTAM, WFDC5, LHX4, SPG7, OR4S2, AHRR, FAM22G, CBX1, SHOX2, PRPF38A, SLC22A6, CSF2, KCNG1, OR5L2, CORT, PRCC, CDY2A, GPR88, ALDH3B1, KRTAP1-1, TUBGCP5, TMEM18, CCR6, CHDH, KRT3, DCST2, C1orf95, C17orf77, GPHA2, C1orf150, STARD7, CNTN1, PGC, CA12, KBTBD8, SPRR2A, NKX2-5, SGIP1, ADA, BTNL9, NCKAP1, CCDC48, DAZ1, FANCM, UNC50, OR5AC2, TRIM11, SLCO1B3, CYSLTR2, CDH6, MAGEB6, DPYS, MORF4L1, KRT24, POLE3, CNBD1, CXCL6, EFCAB2, INS, ROR1, OR3A3, HIST1H4F, SAA2, ANGPTL1, MAK, ESX1, FUT11, ESPN, CST2, CD40LG, DAOA, DHFR, DNAH11, C11orf40, OR4C11, PGLYRP2, PHKG2, CGGBP1, E2F8, NMB, OR2B11, WISP2, SLC35B1, IFITM5, EFCAB1, HIST1H2AA, KGFLP1, OSR1, METTL3, CCL1, NEK7, HIST1H2BA, CDCP2, KLK9, SLC30A6, DDX53, OR5H15, C1orf168, BLOC1S3, LTB4R, XRCC2, RERE, OR2H2, ZBED4, DMP1, SCML2, CCNL2, ADAMTS7, DEFB134, FGF6, ROS1, FLG2, OR5C1, SLC1A7, FOXN1, MSI1, GC, ACCN5, RPS6KA6, MS4A12, PAPD4, FGFRL1, HTR4, WBSCR16, ZNF44, CPA4, TMEM178, IAPP, NOXA1, ZNF200, TMPRSS13, CABP5, OR52E6, HIST1H3A, ZNF271, ITLN1, FAM24A, DMRT3, C6orf174, LCE3A, LOC441177, C14orf126, CASP8AP2, ADAMTS20, KRTAP4-4, FBXL17, KRTAP4-12, HOXA11, INSC, TEX9, NKX2-3, ANKRD1, MAGEA4, TBX18, MMP19, OR4P4, POU6F1, CDH17, TRPT1, SSX3, OR52B2, COPS2, MEOX2 |
| green | HTRA4, HLA-DQA2, HPSE, PLP1, RASL11A, ST8SIA2, LCN9, PDIA2, TSNAX, KIAA1161, CSNK1A1L, VAMP1, POU2F3, SPATA5, UCK2, AQP5, OR10A7, MVD, ITFG1, CHRNA9, TNFRSF10A, BAIAP2L1, IL1RAPL2, NPB, STATH, ACVR1C, HMMR, RPS28, TLX2, SRP9, SERPINA11, KIF1C, IRAK2, ID2, HOXB7, PARS2, KRTAP15-1, KLHDC8B, ASB15, TNFAIP6, MS4A3, SNX9, PAX9, SRPK1, SLC16A11, SCGB2A2, WFDC1, S100A8, IL3RA, ADAR, LYPD2, SLC7A11, CCKAR, VRK1, SIGLEC5, LY6G6D, PLD3, ZC3HC1, HIST1H4D, C10orf68, LGI1, BCMO1, MC2R, ZNF79, OLFM4, DGKK, ETV2, CLDN16, KLF9, XKR7, ZNF101, MADCAM1, IFIT5, UPF2, ACSM1, LEFTY1, OXR1, OSGEP, KIF26B, ASIP, GIPC2, SERPINA9, ZNF586, KCNG2, DEFB114, PHB2, ALDH1L2, PDZRN4, MINK1, HTN3, OR10Q1, SOAT2, POU2F1, RPL37, GPR82, AP1S3, CCL17, FOXM1, GALNT5, SERINC2, CPA1, KRTAP19-2, E2F2, LIG3, OR51A4, ICT1, PNN, OR52E8, PYGM, HIST1H4L, RNASE1, MXD4, CIB4, ITGB1BP3, GCNT3, SERPINA1, NLGN1, TCL6, CAV3, KCNE2, LIPF, AMPD1, C1orf114, SAMD11, PRSS21, TMPRSS5, HSPA14, VWF, TYSND1, SPOCK2, ANKRD30A, DEPDC1B, ADAMDEC1, C1QTNF7, EMR1, CKM, SNHG8, OR4X2, NFIC, LDHAL6A, EMR2, ITGA4, S100A12, MECP2, PRAMEF4, ZNF26, OPTC, HIGD2A, HEY1, NYX, PXN, ABCA13, TIMM44, KLRG2, TYK2, MFGE8, MAGEB4, OR4D5, CCNB2, LAT, AGTRAP, METTL5, FGG, ASB9, TMPRSS6, LAMA2, FLJ40453, PCDHA10, ZNF214, KIR3DL1, NPIP, STK16, VHL, MAGEA10, OR4D2, RABL2B, OR10P1, ATP6V1C2, GATA6, S100A13, AMHR2, RPS23, RNASE10, INGX, CRHR2, ACPP, CHST4, LCE1E, OR5F1, LMO4, FGF10, RARA, ZNF610, PEX13, H2AFB2, HIST1H1B, KRT40, SPATA16, IL22RA2, STARD3NL, IL5RA, GUCY2C, UMODL1, TM7SF3, SERPINE1, PPP3CB, AGBL1, KRTAP19-6, TPRKB, GABRR2, DUSP21, EIF1AY, GPR20, USP47, PKNOX2, BGN, ZNF273, ACOXL, CYP17A1, KLF15, USP6, MSI2, NR2F2, LOC388882, MLF2, OR13C4, VRK3, MC5R, CASP12, MGMT, SLC10A3, SLC1A2 |
| magenta | TRPM2, FA2H, GNAI1, TRIO, TMEM125, LRRFIP1, SPOCK3, ENPP6, HAPLN2, EML1, GJB1, NIPA1, RHBDL2, CD276, RAB33A, CNTN2, KLK6, PLCL1, CNDP1, C4orf32, MRAS, BCAS1, ASPA, CAPN13, ENPP2, CA14, ZNF536, SLC12A2, C18orf56, SYT11, CCNE2, PKP4, CMTM5, GCLC, C11orf9, DNAH17, DBNDD2, CNP, SLC5A11, CTNNA3, GPR62, PCCB, ITGA2, LANCL1, TRIM59, ADI1, SLC13A3, ABCA2, SNX6, GLDN, HOXC9, MOG, RHCE, MAL, SEMA3B, SPOCD1, PTGFRN, RBP1, MAG, AGTPBP1, TYMS, ELOVL7, CDH1, POMGNT1, COL22A1, HMBS, ADARB2, ATG4C, GPR17, PPP1R14A, IL10RB, DLG1, NSUN6, F13B, JUP, CYP2C9, DEFB110, PARVA, EVI2A, FXYD4, ADAMTS14, HS3ST1, HOXD4, CYB5R2, DYSF, TMOD2, GPD1, ANK3, NPR2, HHIP, WDR5B, CLCN1, GOLGA7, CLCA4, CHRM5, CCDC54, COL12A1, RASD1, ENOSF1, GALNT6, MYH3, TCEB2, C9orf24, UEVLD, IVD, HRASLS |
| pink | PLEK, APLP2, FBXO30, LRRC39, TXNDC11, HSD17B12, PRKCZ, GABARAPL2, KIAA1598, CCNJ, LAMA4, PPM1G, ANKFN1, TDRD9, SELM, ASB14, DAAM1, CYS1, STXBP2, NANOS1, ANXA4, TXNDC5, SENP2, DMXL1, ZNF649, CLCN3, STMN4, OR4K2, TMEM123, VAMP5, SLAMF9, GNA13, TRAFD1, OR10X1, ZNF195, DOCK7, CCDC85A, CDC37L1, ARMCX1, TNIP3, ST6GALNAC4, ZNF700, PDE4B, LIFR, CYP7A1, C14orf45, FGF16, GABRG1, LYPLAL1, CREM, BTG3, VGLL1, ALG11, SMAD5, TPP1, PNMT, HHLA3, C6orf120, KCNJ8, KIAA0913, OR51B6, ZBED3, THAP4, DAZ2, TP53I13, NAT9, BTNL8, FAM81B, ACADL, SCGB1D1, ARL4A, TCP11L1, ZNF224, USP8, HSPD1, ADAM30, KRTAP5-2, RAB4A, PDK3, AANAT, XKR3, PMP2, XKR5, COL21A1, PAICS, CXorf36, C1orf192, DEFB126, DAZAP1, ISCU, SMAD2, OTOA, OR52B4, C12orf48, LRRC34, DNAJB7, RGS2, LSM2, OR56B1, C10orf107, UNC45B, LFNG, AGXT2L1, SPAM1, SERPINE2, C11orf36, SPHK2, IQCE, NPY6R, BRWD3, EVX2, PEX7, MYEF2, PSG1, BNIP3L, RAMP3, PDGFD, TSHR, ICA1L, OR4N5, LRFN3, C15orf26, CCNH, RASIP1, IGBP1, SLC25A28, TJP3, CTHRC1, PPP2R5A, ADAMTS5, EPB41L2, RFC3, DNAJB4, CTNS, TPP2, ECHDC1, WDR72, TREML2, MAB21L2, PSMC2, RBM18, SPINK7, NUDCD1, ZNF599, OR4K15, RRBP1 |
| purple | TF, TUBB6, GNG8, SLC5A2, OR7D2, MME, C7orf33, SPAG4, MDFIC, DEFB118, DSC3, FCER2, GOLGB1, GIPC3, OR6K3, TAS1R1, SAMD8, PLXNB1, ENAM, MYB, OR56A4, PHPT1, H2BFXP, KIAA2026, PRO0628, SERPINB7, OR2J2, OR8B12, AQP10, KRTAP3-1, PEMT, B4GALNT3, GALR2, MYBL2, OR11H4, C10orf120, LECT2, SHBG, OR51S1, OR2M2, PDRG1, MYCL1, FAM71A, FAM47A, PEX3, CSTL1, FAM19A3, OR6B1, ARF6, H2BFWT, KCNA10, OR4N2, GOT1L1, TMED8, C1orf187, EFNA2, OPRM1, GJB5, GZMB, PCDHB12, OR7C2, MAGEA11, VKORC1, OR6C76, GLYATL2, MAGEB2, PRND, CXCL10, FUT2, EMR3, CBLC, HSD17B13, DSC1, FRG2, OR9G1, ASB17, SLC17A5, CYP4B1, ALDH1A1, SPINT4, GPR157, OR8A1, RPUSD1, MMP27, UPB1, ARR3, PGM1, CASP14, ZNF721, OR6B3, AVPR1B, SF3B5, GPR55, ZNF551, TBX15 |
| red | OMG, SERPINH1, AQP9, GEM, FLNA, BCL6, TNFRSF1A, PCDHB2, GPX3, LDLR, ZNF460, SPR, CLCF1, GFAP, NDUFS2, LOC441956, MBTPS2, ZNF238, IFITM4P, SAMD4A, ADAM8, IFITM3, GPR135, FBXO42, IFITM2, CCT6B, ZNF550, BAHD1, YIPF1, BVES, ACY3, TGFB1I1, COCH, MAFF, IFITM1, CD86, DNAJB1, CSDA, LYST, KBTBD3, CPNE1, G0S2, TNFRSF10B, BAG3, C6orf145, C21orf59, RBPMS, SFN, OSGEPL1, GADD45B, CPA3, COL4A2, FAM122A, SRCRB4D, PEX10, GYPB, RLF, PDCD6, FKBP5, UBE2J1, GADD45A, ADAMTS18, MT1M, KCNJ16, SRXN1, TRPC4, C10orf54, PRKX, HEYL, TARP, C12orf28, OR6F1, C10orf10, DHRS9, CDKN2C, HTR7, MYADM, FUT7, RBM6, LCE2C, OR1K1, MAGEA6, OVCA2, C1orf38, CIRBP, SIAH2, DNAJA1, SLC9A8, HAMP, ZNRD1, ADAMTS9, NUDT21, IFNA4, COL4A4, SLC3A2, VEGFA, SLC2A14, ZRANB3, LCMT2, GJB4, ZNF658, AZGP1, HSPA1A, MT1G, RSRC2, MT1E, C4orf17, CHORDC1, DMRT1, SLC16A6, CHURC1, OR2B2, C21orf67, PACSIN2, HSPA1B, NR4A3, SPATA6, MYCT1, SARS2, HERC2P2, SARS, F13A1, LEF1, ADM, CDKN1A, HBE1, SLC7A1, MT1F, FLT1, PPP3CC, CKS2, HIST1H4J, MT1B, CRYBB2, GYPA, PGBD4, ZNF433, MYO9A, ZBTB24, BACE2, CPLX3, HINT2, SPAG7, MT1H, TUBB2B, FOSL2, TCP1, ZNF691, DDIT4, FAM83G, THBD, OR2C1, RCOR1, NHLRC1, PRAMEF1, FBXW12, COL4A1, OPHN1, ANGPTL4, MCPH1, TM4SF1, P4HA2, ANKRD37, HSP90AB2P, SSU72, PRDM10 |
| turquoise | CCL18, CFD, APOE, ACP5, FCGR2B, GPNMB, CD68, ALOX15B, APOC1, DENND2D, FBP1, IFI30, HLA-G, FAM20A, PLA2G7, LILRB5, MSR1, LPL, TNFAIP2, PLAU, HLA-DQA1, MAFB, CD300LF, MS4A7, FCGR2C, LTA4H, LGALS3, CXCR4, HLA-C, CCRL2, FCGR2A, TNNI2, GYPC, IL10RA, LILRB3, EPSTI1, LCP1, SQRDL, IFNGR2, ALPK3, HLA-F, BTN2A2, DAB2, CYBA, SERPINF1, SLC16A5, GZMK, EMP3, ARL11, BATF, P2RY8, SAMSN1, HLA-DRB1, ABCA1, LILRA3, HLA-DPB2, IRF8, HLA-DMA, CD33, HLA-E, CD48, HAVCR2, LYN, HLA-DMB, ARHGDIB, CD53, IFI44, PARVG, LILRA4, BST2, CTSC, BTK, MS4A6A, HLA-DPA1, PAQR6, PPARG, HLA-DRB5, LSP1, MGST2, S100A6, IRF7, ADORA3, RGS1, IGSF6, CHI3L1, CPVL, C3AR1, A2M, CAMP, PPT1, MS4A4A, FCER1G, EVI2B, DOK1, CASP4, CMTM3, OLR1, GZMH, LY96, HLA-DRB3, XAF1, IL18BP, C10orf125, C1S, ARHGAP18, MST4, LILRA1, SLA, LAPTM5, FLI1, COLEC12, COL8A2, CXorf21, IFI44L, LILRB1, DNAJC6, ALOX5AP, CD84, SWAP70, CD37, C3, SLFN11, DNAJC5B, LDB3, PIK3CG, ISG15, MYCN, CFI, HLA-DPB1, ANXA2P1, CD74, C1QC, GPR65, VIM, TLR5, FAM46A, IFI16, ARPC1B, REPS2, COTL1, STK10, C1QB, OBFC2A, FAM73A, MBP, SPIN2B, LGALS1, CSTA, ARHGAP30, S100Z, B2M, HLA-A, CASP1, LIPE, MR1, HLA-DQB1, ADPRH, HCLS1, NAGA, RIN3, DOK3, MGAT3, GZMA, SCIN, ZC3HAV1, BIN2, GPSM3, C1R, FAM3B, RGS10, TLR2, PAFAH1B2, IL4I1, ANXA2, AEBP1, TAGAP, TGFBI, FYB, HMHA1, CCR5, PACS2, INPP5D, CLIC1, ADAM28, PLEKHA4, CCR1, ANXA1, PTPN22, OSTM1, CD163, OLIG2, VSIG4, DLEC1, RPL7A, ADORA1, PLCE1, ITPR2, ITGAL, GUCA2B, PRKACB, RNASET2, LRRK2, SLAMF7, TNFAIP8L3, TBXAS1, IL18, TIMP1, PRDM1, SERPINA3, TLR3, TPST2, HGF, YPEL1, MAOB, NFAM1, SIPA1, TCEAL7, THAP10, ITGB2, CCL3, PLSCR1, ANG, LAIR1, C1QA, HCST, CXCR6, IL13RA1, NMI, LMO2, GBP2, DTX3L, TREM2, APOC2, COL8A1, CD2, PYGL, TPST1, SERPINB4, VCL, PCBP4, UGCG, APBB1IP, UBE2S, S100A10, CABYR, HHEX, P2RY6, PALLD, TGFBR1, C10orf116, CHI3L2, CASP5, S100A2, ARHGAP27, MYD88, GDF10, KIF25, CCL3L3, C5AR1, FSD2, PLXNC1, KCNN4, SLC35F3, HBD, OLFML3, TLR7, IGSF8, HIP1R, GNLY, GAMT, PLCD1, ST3GAL6, TMOD3, HS3ST5, B3GNT5, ANKS1B, SLC35B2, SLCO2B1, GIMAP1, CH25H, CLPS, GIMAP2, S100A3, ZFP106, CXCR7, HLA-DQB2, EOMES, CD300A, CP, MMP2, C21orf62, PPP1R16B, GLRX, MYH7B, RBMS1, JAK2, CD14, HLA-DRA, SMAD3, LTF, TMEFF2, CPA6, SRGN, MAPK4, CD44, NCOA7, P2RY2, MYO7A, SOD2, MICAL1, TGIF1, TPPP, PRAM1, IFI27, TNFAIP8L2, ARPC2, NKX6-2, TSPAN6, BLNK, FOLR2, FOXD3, C8A, TTTY21, ZHX1, SIGLEC9, LIMK2, PRKAG2, ZAK, PLCXD3, FAM9A, SOCS6, SFRP2, MBNL1, IRF1, PSCA, SLMO1, FSTL1, HBB, ADAMTS4, N4BP2, WNT5A, LILRA2, RAB11FIP4, RAB13, DDR2, MGST1, IRF4, CNN3, RPA3, ITGB5, SPP1, CCL2, CLEC4M, PSENEN, SERPINB1, PLSCR4, ARHGEF5, CYR61, SLC39A12, LMTK2, TULP4, CYBB, IGFBP7, NRIP1, ASB16, LPIN2, KIRREL3, ZNF114, HLA-DRB4, ZNRF4, COL9A3, GPD2, GREM1, RRAGD, UBE2G1, TGFB1, LAPTM4A, IL33, HTR1A, FCGR3A, RRAS2, ZNF575, EFEMP1, SYTL3, HNMT, FABP7, C8orf4, HPS6, FGA, FAM46C, C1orf162, LAYN, XBP1, PRCP, CHST11, TP53I3, PROS1, PCYT1B, CAPZA3, HIST1H1D, FGL2, HORMAD1, CREBL2, LY6H, TGFB2, PAM, FOS, DPRXP4, TGFBR3, CSAG2, FLG, ASCL4, FABP5, PPIB, TTC8, SIRPB2, CNTNAP3, GPR98, HOXA13, FAM108A1, RAB34, CD302, SLC2A10, CFC1, INSR, TNFSF9, TPM1, BBOX1, FAM105A, BTN3A2, OR7E91P, GALNT1, MTX2, F3, MRGPRX3, SGTA, SLC22A3, FAM70A, GPR34, ITGB3, FOXJ1, TRPV6, FUT3, NXPH2, C9orf140, TLR4, EMP1, PXK, TLR1, TNFRSF11B, UROD, AMIGO2, RHBDF2, CSF1R, IGJ, ANGPT1, AKR1C3, FTCD, HLA-DOB, HBM, HIST1H3D, ZNF483, FCGR1B, ZFP28, KCNH8, FPR1, CETN3, WWC1, ASZ1, GPR77, AQP4, FAS, MRVI1, TCEB3, TLE3, DTNA, IQCF1, DYNLT3, CLEC3A, CD69, GIMAP7, USP20, STOM, GAL3ST4, SYNPO2, SIRT2, SLC9A3, GBP1, TRIB3, LOC401286, PFKL, SLC6A8, LTBP1, RNF126, SLC44A3, SLC16A9, C12orf61, DKK1, NKD2, SLITRK1, RNASE13, GJB6, EBI3, LMBR1, PROCR, PLA2G4A, CLCA2, INPP1, PRH2, NDUFA12, MLLT6, DRD4, WFDC12, CASP7, E2F1, ZNF667, SDHC, FLJ35776, S100P, PTS, FNBP1L, PDE4D, INHBB, DEFB136, PGM2, HBA2, ENPEP, NEBL, BCAS2, RYR1, WEE1, IL2RG, MFAP1, TTL, SLC4A9, PTPRZ1, PGAM2, SPA17, HIST2H2BF, DNTTIP2, PDCD1LG2, MUC17, SACM1L, MCAM, OR4Q3, AGT, DAZL, HOXB6, SPCS3, GPSM1, C15orf48, SLC1A6, DEFB112, CD109, EYA1, HBG1, OTX1, SCG2, KIF3B, ARRDC5, VCX2, KLHL13, TSLP, CYTL1, KRTAP5-7, TRMU, C1orf141, SLC4A7, NAALADL2, NAGS, RBMS2, RAB3IL1, TLN2, TCEAL8, CDCA7, CXCL5, KCNK10, C9, ACSS2, SOX17, CER1, KCNK13, HCG4, MTX1, BMPR1B, CATSPER4, SLC35F2, VAMP2, RASAL1, P4HA1, GMPR, ARFIP2, C1orf87, SLC12A9, AGRN, PRKG2, TBC1D23, CRYGN, ZMIZ2, GPR125, CDX4, KLK7, USH1G, PRMT6, CD226, C15orf32, HK2, TNNI3K, ZNF629, GRM6, RNF180, OR10S1, IL20RA, PCDHB4, OR7D4, SPRR1B, PSG8, DGKE, C20orf166, PNPLA4, SORT1, PIM2, REEP6, KCNE4, CATSPER3, TXNRD2, FLJ46257, OR4A5, FREM1, TLN1, BRD8, PLA2G12B, AQP1, RGN, TRADD, NEK11, GSTT2, CSN2, GFI1, ID3, ANP32E, PLAG1, TSNAXIP1, SLC15A4, ROBO4, FAM72A, RANBP3L, LGALS12, PHYHD1, OR10J3, CDSN, CLDN6, NSUN5, MTSS1, C1orf201, WNT10B, OR12D3, SLC6A14, COLEC10, DYRK3, WDR45, FGB, MYF6, TTR, ZNF696, PLD4, ANKS6, AR, C10orf55, OR2M5, PTGDS, RABL2A, USP15, CST6, HIRIP3, SUSD3, IFNA10, NTNG2, SLCO1C1, TIGD6, OR2S2, WWP1, CTAG1A, HIST1H4C, DBX1, MTHFD2, ZNF248, IL1A, LOC401357, GJA1, NFATC4, TRIM71, SNHG10, POLRMT, SLC5A9, H2AFB3, GABRQ, TMEM9B, CHST5, SPRR1A, DDIT4L, LDHC, MNS1, XPO7, OR10A5, SSX5, POLE4, TRIM52, MYEOV, MEIS2, INTS1, ATP2A1, IFNA16, CTCFL, TNFRSF17, CDRT1, RFX2, ADRBK1, OR6W1P, ZNF668, KIF15, RNASE3, HSPB8, C3orf65, SLC3A1, KLHL12, DEFB121, TTTY14, KLF17, CYP4F3, GPR115, SMTN, C10orf25, OR6C74, KATNB1, USP46, GNRHR2, C21orf58, ADCYAP1R1, IFIT2, RFNG, OR8K3, DMRTA1, ZNF713, ALOX12, PRMT2, TRIM10, PRDX3, SCRN2, COMMD6, OR1J1, TOMM70A, FCGBP, LY75, EDN2, OR5D16, CCL11, DNAH9, ZNF444, P2RX3, TAF1C, ZFYVE28, GPR112, TRIM55, MGAM, TSEN54, UGDH, KLK11, OTOF, S100G, GIPR, BIRC5, DEFA6, PLEKHA3, RAB11FIP3, OR7A17, SNIP1, LYPD3, GPR111, KIAA1919, MLXIPL, TM2D2, PMVK, TTTY8, IFNA14, HGS, ZNF317, GSG2, CDX2, MT4, C9orf47, ZNF2, SERPINA13, SLC39A13, OR52N5, C17orf66, LRRC23, COMP, GRPR, OR52W1, LRRC19, TCTA, ASF1B, TTF1, ANKRD45, WDTC1, OR5M8, DUSP9, WDR60, HSPA1L, TIMM17A, CD38, CALCRL, CCL24, C9orf153, ATG4B, BRAP, MELK, CREB3L3, ZC3H3, ARRDC2, CHRNB3, KRTAP10-8, RBMXL1, OR6S1, CA4, IFT74, KRTCAP3, PHLDA1, RNASE2, FSCN2, GPR150, OR2T5, TMC2, OR6A2, GPR25, IL26, OR5T2, KRTAP10-10, ANKRD22, GRAP2, GHRHR, RPS6KB2, SCRIB, GALR3, MKRN2, PIGV, OPTN, ZBTB16, SCGB1D2, SERP1, LOC441242, UCK1, MTCH2, KRTAP10-12, ZNF324, OR51B4, SRA1, C15orf2, FBXL13, TARBP2, MMP7, MLLT3, SPRY2, OR7G2, CX3CR1, ZNF526, CD164L2, TNK2, TEKT4, C20orf144, CRABP1, RPS4Y1, SLC10A2, MMP10, KRTAP5-3, RPL7, KRTAP6-3, CRISPLD1, LUM, THRAP3, OR5K1, GCNT1, CTSG, CCDC38, CLDN14, ZNF485, UTY, RPS4Y2, SAP130, GPR148, METTL7B, CMTM2, DDX3Y, SCAMP3, KCNE1L, GPR152, MTIF2, FBXO5, UBA52, IL1B, LYSMD4, FBXO36, UBE2A, ZNF703, LOC338799, OR10G4, AKR1C1, CLEC5A, MMP23B, H1FOO, KRTAP5-4, YIPF3, LOC401296, RAB1B, ZNF561, HSF4, UBE2L3, MAFK, ZNF259, DFFB, CPB1, CLDN8, SMAD1, TLR10, RAB5C, ALS2CR12, RHOT2, C20orf151, TNFRSF13B, RHOBTB3, RAB8B, EDNRB, AMN, BUB1, POR, TPR, COL4A6, MCM8, HOXA10, OR9G9, PSPH, C11orf70, DKK4, PLA2R1, GPR84 |
| yellow | SCG5, TEP1, ABCA8, HOXD1, RHOH, CHRM4, LDOC1L, IL2RB, NOV, MUM1L1, CD3G, CPA2, RANBP6, CRTAC1, TMEM116, GBA3, CCR7, SCCPDH, LCAT, LRRC7, DMBX1, SGTB, IL18RAP, PTDSS1, C9orf11, PTGER4, TBL1X, STT3B, RASL12, NEFM, MMP14, CCL13, IGSF1, HMGN2, NEFL, SPIN1, MLH3, PRC1, IGSF21, WWTR1, FGFR2, POP7, SCG3, SLC25A5, OCA2, ZNHIT3, PCSK2, SV2B, SEMA6B, OR1L8, RAB3A, TNFRSF13C, PVALB, OR51E1, SYNJ1, HIST1H2AM, KCNH2, EN1, DDX43, SYT5, PPP3R1, BTG4, STXBP1, SYT3, RIMS2, PPRC1, SERPINB6, TSPAN7, MAGEB1, KRT17, ZBTB7A, OR5V1, LCE3C, ABO, LRRC4C, NRGN, BMP8A, PTK2B, PCDHA3, OR5K4, RNF157, CR2, OR6V1, GPR174, ZIC1, DLGAP4, OR2A14, PCYT1A, GALNT7, NPM2, SNAP25, SCMH1, AOX1, RCHY1, ARSI, TOMM40L, KRTAP13-4, SUMO1, TRRAP, AGPAT1, KIF6, NELL2, SVOP, C4orf7, TRPC7, DSCR9, SH3GL2, OLFM1, MDH1, VAV3, INCENP, SMEK3P, FGF3, SYT4, LIMK1, SYN2, SPTBN2, GPR151, SCAMP5, C15orf33, TM4SF5, RPH3A, MPPED2, ST8SIA3, LHX2, CYP11B1, POU3F1, OCIAD1, TACC2, OR2M3, OR10H5, TBC1D22A, SCN2B, HS6ST3, SLU7, TRIM56, VPS72, CST5, OPCML, PSEN1, TEKT1, RYR2, FPGT, LOC283392, RASSF8, PFN2, OAF, SGCZ, INHA, PRDM12, HOXA4, ZNRF1, SLC12A5, OR7G3, GANC, SLC4A3, HES2, RAD23B, SUB1, KCNJ3, MRAP, ENAH, OR1J2, OR2T1, LEMD1, HAO1, AGR2, C11orf65, LRPAP1, KIAA1522, ADRBK2, MCTP1, LRRC36, MLLT11, IKBKAP, FBXL5, SPAG9, VSNL1, NOS1AP, CHRM2, BRDT, MAST1, PRODH, PHLDB2, ABCD2, SLC8A3, CCDC34, ARC, PNMA5, FOXP3, MARK1, PFN3, UNC5D, LIMS2, ITIH5, KLF3, SPATA20, LOC407835, SIRT5, NAPB, STC2, REEP2, HOXB8, BBS9, OR2T10, MTERFD2, TSPYL1, CD207, GNGT1, PART1, PHACTR3, OR5M11, SRRM2, KRTAP5-10, CSF3, TPO, SYT7, EML5, PCDHA4, TM9SF4, KEL, RASA4, AQP7, RBM5, PIGQ, AGBL2, SLC44A5, CCL8, IFIT1, SLC9A6, ZNHIT1, OR5M3, WDR37, PCDHGA7, ZNF532, PLK2, WNT2, YES1, TLE4, ITGBL1, ITPKA, MYLIP, PDHA1, XKR4, NPY, HIPK3, RAP1A, OR5M9, OR5AR1, PRKACA, HIST1H2BC, AGR3, GML, STMN2, LOX, AKAP5, PAXIP1, SYTL2, ITIH2, GRIN2D, CGB2, OR5J2, UTP15, LONRF2, MMP17, SLC26A8, NUP214, NPAS3, MON1A, HOXC11, NPDC1, FAM92B, PNKP, DST, L3MBTL2, CYP4F2, PNKD, GPR158, ZNF274, SLC9A7, PCDHA8, FAM53A, FZD6, DSG1, CLEC4D, HOXB2, HIST1H2AJ, MAGI3, SLC30A8, HIST1H1A, PTPN11, ZNF346, NR1D1, TRIM39, SPATA9, HTR2A, RGS4, GRASP, ZNF345, TCF7, CILP, SPRY4, ART3, HIVEP2, RAB6B, PGM2L1, GPR32, RFC2, C1orf9, XCR1, SIX1, LCE2D, HIST3H3, SHPRH, SMPD3, CYP4A22, OR13G1, PSD3, MGC2752, HSFY2, CYP8B1, FNDC1, KIAA0513, RFWD3, CLLU1, TARS, INADL, OR8U8, XAGE3, MATK, OSMR, MICAL2, PRL, SYT1, PLG, RGS6, SIRT4, HIST1H2BH, OR51Q1, PCSK1, RAB5A |
| grey | ALB |
